# Stable ultrathin partially oxidized copper film electrode for highly efficient flexible solar cells

**DOI:** 10.1038/ncomms9830

**Published:** 2015-11-05

**Authors:** Guoqing Zhao, Wei Wang, Tae-Sung Bae, Sang-Geul Lee, ChaeWon Mun, Sunghun Lee, Huashun Yu, Gun-Hwan Lee, Myungkwan Song, Jungheum Yun

**Affiliations:** 1 Key Laboratory for Liquid-Solid Structural Evolution and Processing of Materials, School of Materials Science and Engineering, Shandong University, Jinan 250061, China; 2 Division of Surface Technology, Korea Institute of Materials Science, Changwon, Gyeongnam 641-831, Republic of Korea; 3 Institute of Hybrid Materials, The Growing Base for State Key Laboratory, Qingdao University, Qingdao 266071, China; 4 Jeonju Center, Korea Basic Science Institute, Jeonju, Jeonbuk 561-180, Republic of Korea; 5 Daegu Center, Korea Basic Science Institute, Daegu 702-701, Republic of Korea; 6 Division of Commercialization Research, Korea Institute of Materials Science, Changwon, Gyeongnam 641-831, Republic of Korea

## Abstract

Advances in flexible optoelectronic devices have led to an increasing need for developing highly efficient, low-cost, flexible transparent conducting electrodes. Copper-based electrodes have been unattainable due to the relatively low optical transmission and poor oxidation resistance of copper. Here, we report the synthesis of a completely continuous, smooth copper ultra-thin film via limited copper oxidation with a trace amount of oxygen. The weakly oxidized copper thin film sandwiched between zinc oxide films exhibits good optoelectrical performance (an average transmittance of 83% over the visible spectral range of 400–800 nm and a sheet resistance of 9 Ω sq^−1^) and strong oxidation resistance. These values surpass those previously reported for copper-based electrodes; further, the record power conversion efficiency of 7.5% makes it clear that the use of an oxidized copper-based transparent electrode on a polymer substrate can provide an effective solution for the fabrication of flexible organic solar cells.

## Abstract (web summary)

Light enters and exits optoelectronic devices through transparent conductive electrodes, which are one of their most expensive components. Here, the authors develop stable transparent conductive electrodes based on copper and oxide layers that lead to efficient flexible organic solar cells.

## Graphical abstract

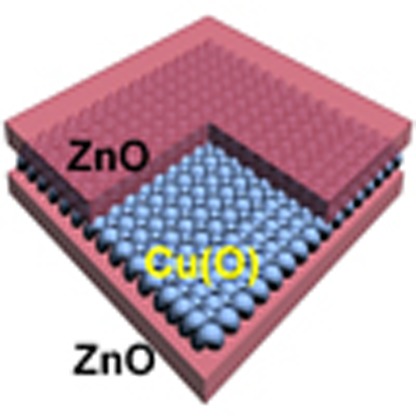

The fabrication of transparent conducting electrodes on mechanically flexible and heat-sensitive polymer substrates is crucial for the realization of the latest flexible displays and photovoltaic devices. A flexible transparent conducting electrode (FTCE) is required to obtain the high electrical conductivity, optical transparency, mechanical flexibility and long-term chemical stability that cannot be achieved using conventional single-film-type transparent conducting oxides such as indium tin oxide (ITO). At present, the best FTCEs are based on an expensive metal, Ag, in the form of nanopatterns^1^, nanowires^2^,^3^,^4^ and continuous films^5^,^6^,^7^,^8^,^9^,^10^,^11^. Carbon-based FTCEs, such as carbon nanotubes and graphene, exhibit optoelectrical properties that are far inferior to those of Ag-based FTCEs^12^. Considering that FTCEs are one of the most expensive components in optoelectronic devices, it is important to replace the currently available Ag-based FTCEs with an alternative that is based on a low-cost electrode material.

Cu has received much attention as a promising candidate for replacing Ag within FTCEs because of its low electrical resistivity (1.68 μΩ cm at 25 °C)^13^ that is comparable to that of Ag (1.59 μΩ cm at 20 °C), and its competitive cost, which is ∼100 times less than that of Ag (ref. 14). Among the various possible Cu-based FTCEs^14^,^15^,^16^,^17^,^18^,^19^,^20^,^21^, optimal optoelectrical performances have been achieved using Cu nanowires with transmittances of 80% at sheet resistances of ∼20 Ω sq^−1^ (refs 14, 15, 16, 17, 18, 19). However, these values are far inferior to those of Ag-based FTCEs that exhibit transmittances greater than 90% at sheet resistances much lower than 20 Ω sq^−1^ (refs 3, 4, 10). In addition to the high propensity of Cu to oxidize under ambient conditions, the unique structure of Cu nanowires, with large surface areas and nanoscopic diameters, triggers oxidation on exposure to ambient environments, which seriously degrades their electrical conductance. There have been several efforts to resolve the oxidation problems in Cu electrodes without any significant accompanying degradation of their optoelectrical properties^17^,^18^,^19^,^22^,^23^. Recently, Wiley *et al*. reported on the protection of Cu nanowire films against oxidation through the electrodeposition of transition metal shells, followed by shell oxidation, on the nanowires^19^. Hatton *et al*. have shown that highly conductive ultrathin Cu films can form when using a molecular adhesive layer, and their oxidation can be passivated effectively by coating with a sub-1-nm-thick aluminium oxide film^22^. However, an effective solution that allows the fabrication of highly transparent and conductive Cu-based FTCEs with long-term stability has not yet been established.

The insertion of Cu between transparent oxide films in the form of a continuous, smooth and ultrathin film in an oxide–metal–oxide configuration could be a realistic approach for use in preventing Cu oxidation by inhibiting the penetration of molecules of oxygen and water vapour via the encapsulation of the Cu by the oxide films^24^,^25^. An oxide–metal–oxide structure that contains a ductile metal layer also provides improved structural durability against serious mechanical deformation when compared with single-film-type oxide electrodes. However, despite the advantages presented by Cu-based oxide–metal–oxide electrodes, these have not been widely accepted as FTCEs because of their poor optoelectrical performance compared with the aforementioned alternate FTCEs^24^. A deleterious compromise between optical transmittance and electrical conductivity is inevitable in the case of Cu films because an increase in the film thickness, accompanied by a strong reduction in the transmittance, is necessary to improve conductivity. Therefore, achieving a completely continuous and smooth Cu film with an ultralow thickness that is far lower than any thickness value ever reported for continuous Cu films is viewed as an essential requirement for the development of competitive, efficient Cu film-based FTCEs. However, because Cu, along with Ge (refs 26, 27), Ni (ref. 26) and Cr (ref. 27) is considered to present excellent wettability on heterogeneous surfaces^28^, further improvements in wettability present a challenge, and no currently available technique provides a solution to this issue.

Here we disclose that a completely continuous and smooth Cu film was produced at an ultralow thickness of ∼2 nm as a result of a significant improvement in the wettability of the Cu that was sandwiched between two ZnO films. This development was achieved through a significant improvement in film wettability owing to the successful suppression of nanoscopic cluster migration with limited Cu oxidation during the very early stages of Cu growth, and without any degradation in its electrical conductivity. We found that coalescence between neighbouring Cu clusters through active surface migrations, known to occur universally during the nucleation and early growth stages in conventional metal deposition processes, was substantially suppressed in our weakly oxidized Cu (Cu(O)) films. The oxide–metal–oxide FTCE that employed an ultrathin Cu(O) film exhibited excellent optoelectrical properties, which far surpassed the best values previously reported for any Cu-based electrode. A flexible organic solar cell employing the Cu(O)-based oxide–metal–oxide FTCE exhibited a record power conversion efficiency (PCE) of 7.5%, outperforming a flexible organic solar cell that used an ITO electrode.

## Results

### Fabrication of an ultrathin Cu(O) Film

To fabricate continuous, ultrathin Cu films, a method is required to suppress growth in the Volmer–Webber mode, which is described as a three-dimensional (3D)-island growth, during the growth of the metal on heterogeneous substrates. Very recently, we reported the possible suppression of the 3D-island growth mode in oxygen-doped Ag films; however, the exact role of oxygen doping in this morphological improvement was not clearly understood^10^,^29^. Here we demonstrate that the abrupt morphological transition from rough and granular 3D films to highly smooth and continuous two-dimensional (2D) films during the very early growth stages is facilitated by limiting the oxidation of Cu films using precisely controlled oxygen doses.

**Figure 1:**
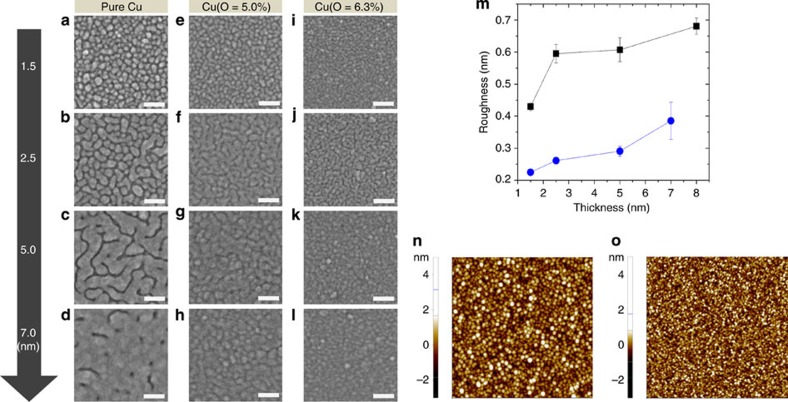
Morphology of Cu and Cu(O) films. Field-emission scanning electron microscopy images showing morphological differences between 1.5-, 2.5-, 5.0- and 7.0-nm-thick (**a**–**d**) pure Cu, (**e**–**h**) Cu(O=5.0%) and (**i**–**l**) Cu(O=6.3%) films deposited on 20-nm-thick ZnO films. Scale bar, 40 nm. (**m**) Atomic force microscopy results showing root-mean-square surface roughness of (black square) Cu and (blue circle) Cu(O=5.0%) films. The solid lines are drawn to guide the eyes. Average surface roughness was determined from the measurement of at least three different scan domains (1 μm × 1 μm) for each sample. Error bars indicate the s.d. Two-dimensional images of a scan domain (1 μm × 1 μm) show the morphology of 5-nm-thick (**n**) Cu and (**o**) Cu(O=5.0%) films.

This morphological transition was observed using ultrahigh-resolution (UHR) field-emission scanning electron microscopy (FE-SEM) (Fig. 1) and atomic force microscopy (AFM) measurements (Supplementary Figs 1 and 2). The oxidation level of the Cu films was controlled by introducing a mixture of Ar and O_2_ gases with various input O_2_/Ar flow ratios during the reactive sputtering process (Supplementary Fig. 3). This was evaluated using X-ray photoelectron spectroscopy depth profiling (Supplementary Fig. 4) and Cu 2*p* peak spectra measurements (Supplementary Fig. 5). The optimal oxidation levels of the Cu films were achieved using O/Cu ratios of 5.0% and 6.3%, represented as Cu(O=5.0%) and Cu(O=6.3%), respectively. This approach ensured that oxygen inclusion in the Cu films was relatively low to minimize deterioration of the electrical conductivity. The continuous Cu(O) films exhibited a smaller percolation threshold thickness compared with that of the pure Cu film (Fig. 1a–l). Ultrathin continuous films of 2.5 nm (Fig. 1f) and 1.5 nm (Fig. 1i) were achieved for Cu(O=5.0%) and Cu(O=6.3%), respectively. However, a continuous pure Cu film was only obtained at thicknesses greater than 5 nm (Fig. 1c–d). The morphologies of the Cu(O) films, which contained much smaller and flatter granules than the Cu film, were effective in reducing the surface roughness (Fig. 1m–o). The root-mean-square roughness was estimated to be ∼0.3 nm for a 5-nm-thick Cu(O=5.0%) film, whereas that of a Cu film of the same thickness was estimated to be ∼0.6 nm.

**Figure 2:**
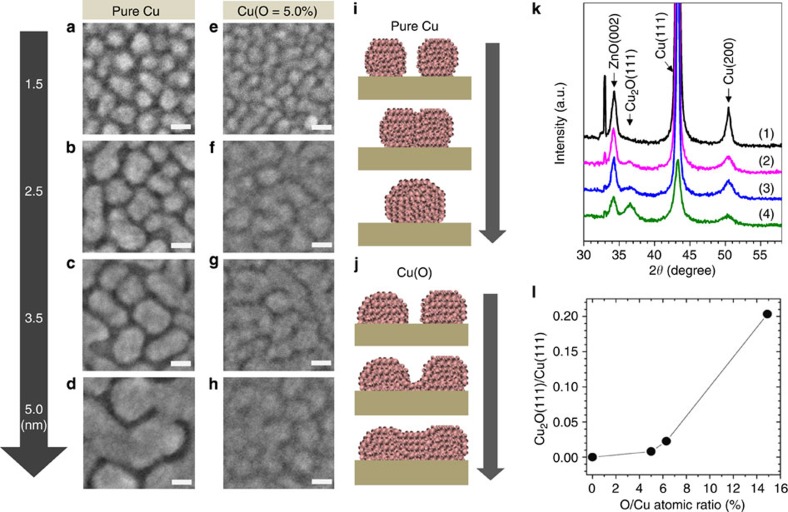
Coalescence of Cu and Cu(O) films. Highly magnified field-emission scanning electron microscopy images showing the coalescence behaviour of (**a**–**d**) Cu and (**e**–**h**) Cu(O=5.0%) clusters with low thicknesses (1.5–5.0 nm) deposited on ZnO films during the very early stages of growth. Scale bar, 10 nm. Conceptual diagram showing the cluster coalescence mechanism of (**i**) Cu and (**j**) Cu(O=5.0%). (**k**) X-ray diffraction patterns demonstrating the change in crystallography as a function of O/Cu atomic ratio: (1) 0%, (2) 5.0%, (3) 6.3% and (4) 14.9%. (**l**) Corresponding oxidation levels, as determined by the peak intensity ratio of Cu_2_O(111)/Cu(111). The solid line is drawn to guide the eyes.

The differences in the morphological evolution during the early stages of growth were evaluated in more detail using highly magnified 50 nm × 50 nm FE-SEM images of the Cu and Cu(O=5.0%) films (Fig. 2a–h). The distinct morphologies of the Cu and Cu(O=5.0%) films could be ascribed to differences in the coalescence between neighbouring nanoscopic metal clusters. It is generally accepted that the formation of noble metal films is a result of drastic and uncontrollable cluster coalescence that occurs immediately after saturation of the nucleation. The FE-SEM images (Fig. 2a–h) demonstrate that the early growth of pure Cu was accompanied by coalescence between neighbouring clusters via the active migration of nanoscopic island-like Cu clusters, as described by the well-known process of cluster-mobility coalescence^30^. This cluster coalescence induced a continuous increase in the size of the evolving clusters, accompanied by a reduction in the cluster density. The cluster size of the Cu film exhibited a continuous evolution, even when the film reached a thickness of 5 nm (Fig. 2a–d). Meanwhile, the evolution of the Cu(O) films exhibited a distinct coalescence mechanism, which indicated coalescence via a neck (or bridge) connection between neighbouring clusters, without active cluster migration (Fig. 2e–h). The clusters in the Cu(O) film were connected by irregularly shaped necks, even at a low thickness of 1.5 nm (Fig. 2e). A continuous Cu(O) film evolved at a thickness of 2.5 nm because of the successive growth of the necks (Fig. 2f); this is known to consequently reduce the total surface energy of the cluster geometry^30^. The suppression of cluster migration was confirmed by the cluster size, which was clearly smaller than that of Cu clusters at the same thickness. Once the film formed a continuous morphology at a thickness of 2.5 nm, there were no obvious changes in the cluster size and density of the evolving Cu(O). Schematic diagrams demonstrate the specific differences between the cluster coalescence mechanisms present in the developing Cu (Fig. 2i) and Cu(O) films (Fig. 2j).

It is crucial to note a significant amount of the crystallographic Cu oxide phase (represented by Cu_2_O) was not observed in the Cu(O=5.0%) and Cu(O=6.3%) films (Fig. 2k). The primary phase components consisted of metallic Cu phases with a predominant Cu(111) texture. The proportions of the Cu_2_O phase in the Cu(O) films, determined by the intensity ratio of Cu_2_O(111)/Cu(111) in the X-ray diffraction spectra, were only 0.8% and 2.3% for the Cu(O=5.0%) and Cu(O=6.3%) films, respectively (Fig. 2l). From these results, it is reasonable to assume that the majority of the oxygen occupied atomic sites within the Cu lattices rather than forming discrete oxide phases. Furthermore, the increase in the full-width at half-maximum of the Cu(111) peak as the oxidization level increased provided evidence that the Cu(O) films consisted of smaller Cu crystallites due to the suppression of the cluster-mobility coalescence (Supplementary Table 1).

### FTCE performance

**Figure 3:**
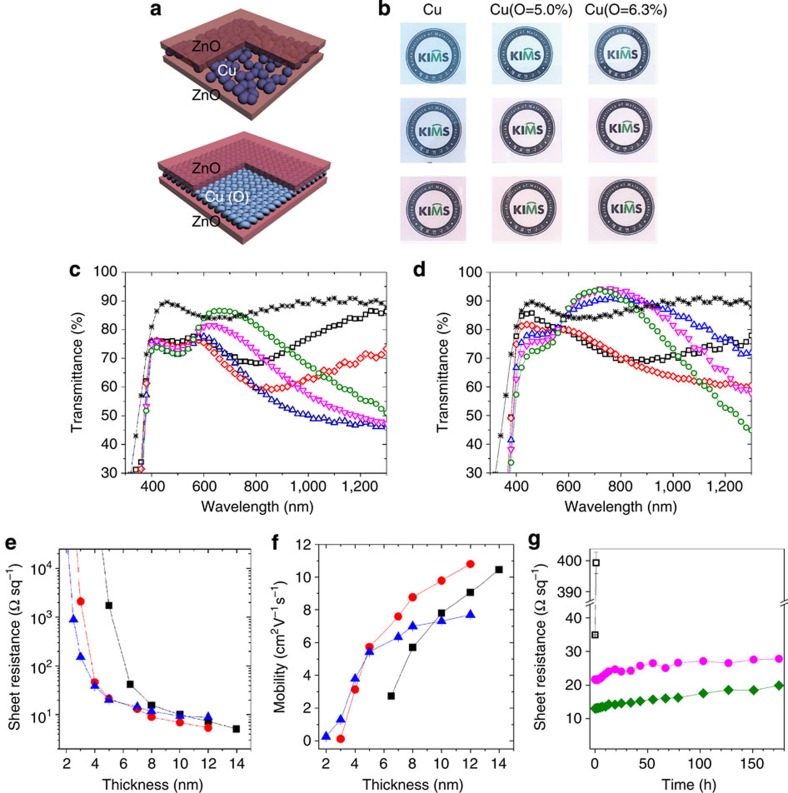
Optoelectrical performance of oxide–metal–oxide FTCEs. (**a**) Schematic diagrams representing the different morphologies of Cu and Cu(O) films sandwiched between 60-nm-thick ZnO films in an oxide–metal–oxide configuration. (**b**) Optical photographs of (1) ZnO/Cu/ZnO FTCEs with Cu thicknesses of 2.5, 5.0 and 8.0 nm, (2) ZnO/Cu(O=5.0%)/ZnO FTCEs with Cu(O=5.0%) thicknesses of 2.5, 5.0 and 7.0 nm and (3) ZnO/Cu(O=6.3%)/ZnO FTCEs with Cu(O=6.3%) thicknesses of 2.5, 5.0 and 7.0 nm. Total transmittance of (**c**) ZnO/Cu/ZnO and (**d**) ZnO/Cu(O=5.0%)/ZnO FTCEs with various Cu and Cu(O=5.0%) thicknesses: black square, 1.5-nm-thick Cu and Cu(O); red diamond, 2.5-nm-thick Cu and Cu(O); blue triangle, 5.0-nm-thick Cu and Cu(O); magenta inverted triangle, 6.5-nm-thick Cu and 7.0-nm-thick Cu(O); green circle, 8.0-nm-thick Cu and Cu(O) films; and black asterisk, 120-nm-thick ITO film. Change in (**e**) sheet resistance and (**f**) corresponding carrier mobility of FTCEs with (black square) Cu, (red circle) Cu(O=5.0%) and (blue triangle) Cu(O=6.3%) film thicknesses. (**g**) Increase in sheet resistance of (magenta circle) ZnO/8-nm-thick Cu/ZnO, (green diamond) ZnO/7-nm-thick Cu(O=5.0%)/ZnO and (black square) ZnO/8-nm-thick Cu films without a top ZnO film at 85 °C and 85% relative humidity. Error bars indicate the s.d. of at least three measurements. The solid lines are added to guide the eyes.

A Cu(O) film that exhibits unique morphological features, such as a completely continuous, smooth morphology at an ultralow thickness, is only an excellent FTCE candidate if its electrical conductivity does not degrade via oxidation. Identical oxide–metal–oxide configurations were employed for both ultrathin Cu and Cu(O) films embedded between 60-nm-thick ZnO films (Fig. 3a). Optimal optoelectrical properties were obtained in the case of the ZnO/Cu(O=5.0%)/ZnO FTCE, and non-negligible degradations in both the electrical conductance and optical transmittance were observed for the ZnO/Cu(O=6.3%)/ZnO FTCE (Fig. 3b and Supplementary Table 2). However, the properties of the latter electrode were still superior to those of the ZnO/Cu/ZnO FTCE. The ZnO/7-nm-thick Cu(O=5.0%)/ZnO FTCE exhibited an average transmittance of 85% in the visible spectral range of 400–800 nm (Fig. 3d) and a sheet resistance of 13 Ω sq^−1^ (Fig. 3e). When the Cu(O) film thickness was increased to 8.0 nm, the ZnO/Cu(O=5.0%)/ZnO FTCE exhibited an average transmittance of 83% and a sheet resistance of less than 10 Ω sq^−1^. The transmittance surpassed that of a 120-nm-thick ITO film in the spectral range of 600–900 nm. The ZnO/8-nm-thick Cu/ZnO FTCE exhibited an average transmittance of 79.8% (Fig. 3c) and a sheet resistance of ∼16 Ω sq^−1^ (Fig. 3e). Compared with the ZnO/Cu/ZnO FTCE, the improved transmittance of the ZnO/Cu(O=5.0%)/ZnO FTCE was a result of the effective suppression of the absorbance and reflection in the visible spectral range owing to the formation of a superior morphology (Supplementary Figs 6 and 7). The increase in the thickness of the ZnO films also contributed to increases in the transmittance of the ZnO/Cu/ZnO and ZnO/Cu(O=5.0%)/ZnO FTCEs over the visible and near-infrared spectral ranges, although the transmittance of the ZnO/Cu(O=5.0%)/ZnO FTCE was still great for thicker ZnO films when compared with that of the ZnO/Cu/ZnO FTCE (Supplementary Fig. 8). Larger increases in the transmittance with increasing ZnO thickness were observed for the ZnO/Cu (O=5.0%)/ZnO FTCE at wavelengths longer than 650 nm, whereas very similar tendencies in terms of improvements in transmittance with increasing ZnO thickness were observed for both FTCEs in the bandwidth of 400–600 nm. There was no significant shift of the band maxima with increasing ZnO thickness in the light absorption spectral range, 400–750 nm, of the solar cells used in this study.

The optoelectrical performance of the FTCEs was further evaluated by comparing their figure of merits, which is a standard index used to quantitatively determine transparent conducting electrode performance, with that of a 120-nm-thick ITO film. The figure of merit was determined using the expression, figure of merit=*T*^10^/*R*_sheet_, (ref. 31) where *T* is the average transmittance and *R*_sheet_ is the sheet resistance. The figure of merit values between 8.2 × 10^−3^ and 17.7 × 10^−3^ Ω^−1^ obtained for the ZnO/Cu(O=5.0%)/ZnO FTCE outperformed those of the ZnO/Cu/ZnO and single-ITO-film FTCEs (Supplementary Table 3). Since the sputtering process employed for producing the FTCEs is already a standard fabrication technique that is used for coating numerous metal and oxide layers on polymer substrates, the FTCEs can be readily scaled up on a polymer substrate with an increased area of more than 10 cm × 10 cm (Supplementary Fig. 9). A further scale up of the FTCEs is quite practicable through the use of Cu and ZnO targets with an increased size on large-area polymer substrates.

The dependence of the sheet resistance and corresponding carrier mobility of the oxide–metal–oxide FTCEs on the thickness of the Cu and Cu(O) films demonstrated the superiority of the ZnO/Cu(O)/ZnO FTCEs compared with the ZnO/Cu/ZnO FTCE (Fig. 3e,f). The differences in the sheet resistance could be ascribed to differences in the carrier mobility, since the FTCEs containing Cu, Cu(O=5.0%) and Cu(O=6.3%) films exhibited very similar carrier concentration values (Supplementary Fig. 10). The carrier mobility of the ZnO/Cu(O=5.0%)/ZnO FTCE reached a saturation value at relatively low metal thicknesses compared with those of the two other electrodes (Fig. 3f). Carrier mobility was significantly suppressed by the surface scattering that was due to the discontinuous and rough granular film morphology of the FTCE with the Cu film^5^,^32^. However, such scattering was far less likely to occur in the continuous and smooth Cu(O=5.0%) film. The reduction in the carrier mobility as the oxygen concentration further increased to 6.3% could be explained by an increase in the carrier scattering originating from the degradation of the Cu crystallinity in the Cu(O=6.3%) film that was due to a greater amount of oxygen impurities.

The ZnO/Cu(O=5.0%)/ZnO FTCE exhibited long-term stability on exposure to an ambient environment, without significant electrical degradation due to oxidation (Fig. 3g). The FTCE still retained a low sheet resistance of less than 20 Ω sq^−1^ following its exposure to accelerated humidity conditions (at a temperature of 85 °C and a relative humidity of 85%) for more than 170 h, whereas the Cu film, which was directly exposed to ambient air for 2 h with no ZnO film present, underwent electrical degradation. Furthermore, the FTCE remained exceptionally stable against continuous long-term ultraviolet radiation for 170 h in air. No change in the sheet resistance and optical transmittance of the FTCE reflects its high durability against ultraviolet radiation (Supplementary Fig. 11), which was because of the effective ultraviolet radiation shielding provided by the ZnO films.

### Organic solar cell performance

**Figure 4:**
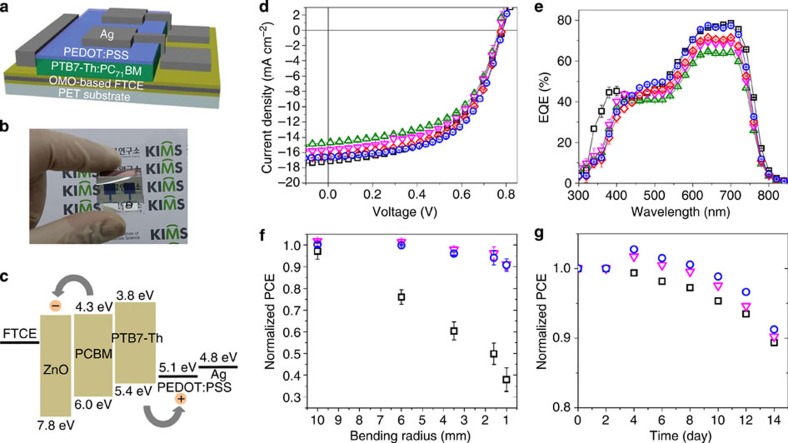
Organic solar cell applications. (**a**) Device architecture and (**b**) optical photograph of the flexible inverted organic solar cell used in this study. (**c**) Schematic energy diagram of the electronic structure of the solar cell. Comparison of (**d**) current density–voltage characteristics and (**e**) external quantum efficiency (EQE) spectra of solar cells using different FTCEs: green triangle, ZnO/6.5-nm-thick Cu/ZnO; magenta inverted triangle, ZnO/8.0-nm-thick Cu/ZnO; red diamond, ZnO/5.0-nm-thick Cu(O=5.0%)/ZnO; blue circle, ZnO/7.0-nm-thick Cu(O=5.0%)/ZnO; and black square, single 120-nm-thick ITO film electrodes. (**f**) Change in PCE, normalized to the initial value, as a function of bending radius during irreversible bending testing of solar cells using different FTCEs: magenta inverted triangle, ZnO/8.0-nm-thick Cu/ZnO; blue circle, ZnO/7.0-nm-thick Cu(O=5.0%)/ZnO; and black square, single 120-nm-thick ITO film electrodes. Error bars represent the s.d. (*n*=5). (**g**) Change in the PCE as a function of time for solar cells using FTCEs corresponding to (**f**).

The high optical transmittance and low electrical resistance of the ZnO/Cu(O=5.0%)/ZnO FTCE resulted in the highest photocurrent conversion efficiency ever recorded for inverted organic solar cells using a Cu-based FTCE as the front transparent electrode. Here the solar cell employed a low band-gap photoactive polymer, which exhibited strong light absorption in the spectral region that extends to longer wavelengths of up to 750 nm. The solar cell was fabricated on highly flexible polyethylene terephthalate (PET) substrates with the configuration: PET/FTCE/photoactive layer/poly(3,4-ethylenedioxythiophene) poly(styrenesulfonate) (PEDOT:PSS)/Ag (Fig. 4a,b). The photoactive layer consisted of a mixture of an electron donor, poly[[4,8-bis[(2-ethylhexyl)oxy]benzo-[1,2-b:4,5-b′]dithiophene-2,6-diyl][3-fluoro-2-[(2-ethylhexyl)-carbonyl]-thieno-[3,4-b]-thiophenediyl]] (PTB7-Th), and an electron acceptor, [6,6]-phenyl-C71-butyric acid methyl ester (PC_71_BM). The hole-transport material, PEDOT:PSS, was chosen because of its relatively high hole mobility and an appropriate work function that coordinated well with that of PTB7-Th in the sequence employed (Fig. 4c). The current density–voltage (*J−V*) characteristics (Fig. 4d) and external quantum efficiency spectra (Fig. 4e) of the solar cells were measured using the 120-nm-thick ITO, ZnO/Cu/ZnO and ZnO/Cu(O=5.0%)/ZnO FTCEs. The short-circuit current density (*J*_sc_), open-circuit voltage (*V*_oc_), fill factor (FF) and the corresponding PCE were determined from the *J−V* characteristics (Table 1). The solar cell using the ZnO/Cu(O=5.0%)/ZnO FTCE exhibited a PCE value of 7.5% at a Cu(O=5.0%) film thickness of 7.0 nm, whereas the solar cell with the ZnO/Cu/ZnO FTCE exhibited a PCE value of 6.64% at a Cu film thickness of 8.0 nm. Furthermore, the highest PCE value obtained surpassed the PCE (6.91%) of the solar cell with a conventional ITO/ZnO FTCE that consisted of a 50-nm-thick ZnO film coated on a 120-nm-thick ITO film.

The improvement in the PCE value for the solar cell with the ZnO/Cu(O=5.0%)/ZnO FTCE was predominantly because of its high FF and *J*_sc_, which were sensitive to the improvement in the electrical conductance and optical transmittance of the FTCE, respectively. The lowest sheet resistance of the FTCE that was optimized with the 7.0-nm-thick Cu(O=5.0%) film contributed directly to the increase in the FF of its solar cell. Moreover, the high transmittance contributed to the increase in the *J*_sc_ as a result of the enhanced absorbance in the photo-absorbable polymer layer. In contrast, the PCEs of the solar cells with the ZnO/Cu/ZnO FTCEs were restricted by their low *J*_sc_ values that were owing to the poor optical transmittance of the FTCEs. The PCE of the solar cell with the ITO film was restricted by the low FF value that was owing to the high sheet resistance (∼60 Ω sq^−1^) of the 120-nm-thick single ITO film fabricated on the PET substrate at room temperature. Although the optical transmittance of the ZnO/Cu/ZnO and ZnO/Cu(O=5.0%)/ZnO FTCEs was further improved by increasing the ZnO thickness from 60 to 80 nm as mentioned above, the improvement in the optical transmittance did not result in an increase in the PCE of the solar cells with these FTCEs. The PCEs of the solar cells with 80-nm-thick ZnO films were significantly lower than those of the solar cells with 60-nm-thick ZnO films for both ZnO/Cu/ZnO and ZnO/Cu(O=5.0%)/ZnO FTCEs. This was a result of significant increases in the *R*_s_ and, thus, reductions in the charge collection efficiencies of solar cells owing to the increase in the ZnO thickness to 80 nm (Supplementary Table 4 and Supplementary Fig. 12). It is also noteworthy that the PCE of the ZnO/Cu(O=5.0%)/ZnO FTCE was maximized at a photoactive layer thickness of ∼100 nm and, a further increase in the thickness, to ∼130 nm in this study, led to a significant reduction in the PCE (Supplementary Table 5 and Supplementary Fig. 13). The corresponding *J*_sc_ and FF values indicated that the enhancement in the light absorbance of the photoactive layer was saturated at a relatively small thickness of 100 nm.

Compared with the solar cell using a single ITO film, the excellent mechanical flexibility and long-term stability of the ZnO/Cu(O)/ZnO FTCEs ensured superior durability for the solar cells employing these FTCEs. When the solar cells were exposed to extreme compressive stress induced by the bending of the PET substrates according to a two-point bending technique (Supplementary Fig. 14)^33^,^34^, the solar cells with the oxide–metal–oxide FTCEs showed an ∼10% reduction in PCE, even after bending with a bending radius of 1 mm (Fig. 4f). The change in the PCE value was much smaller than that of the solar cell with a single ITO film, which exhibited a reduction of more than 60%. This was because of the reduction in the *J*_sc_ and FF values owing to the formation of microscopic cracks in the ITO film (Supplementary Fig. 15). Furthermore, the solar cell with the ZnO/Cu(O)/ZnO FTCE retained ∼90% of its initial PCE value following 15 days in an ambient environment, which is comparable to the long-term stability of the solar cell using a single ITO film (Fig. 4g).

## Discussion

A simple strategy is presented for synthesizing a completely continuous 2D Cu film at an ultralow thickness of ∼2 nm, through the oxidation of Cu using precisely controlled oxygen doses. We found that the limited Cu oxidation substantially suppressed the 3D Cu clustering via cluster-mobility coalescence and resulted in a significant improvement of the wettability of the Cu supported by oxide films. The oxide–metal–oxide configuration, consisting of a 7- to 8-nm-thick Cu(O) ultrathin film sandwiched between ZnO films on polymer substrates, produced a FTCE with optoelectrical features (average optical transmittances of 83–85%, with a maximum transmittance of 94% over the visible spectral range of 400–800 nm, and sheet resistances of 9–13 Ω sq^−1^) and long-term stability against oxidation, which far surpassed the best features ever reported for Cu-based electrodes. The superior properties of this FTCE resulted in an inverted organic solar cell that exhibited a record PCE of 7.5%, outperforming those of organic solar cells employing conventional ITO and other Cu-based FTCEs. This study provides a valuable scientific discovery, which resolves technical challenges related to FTCEs through their fabrication, using the abundant and economical Cu rather than the rare and expensive ITO and Ag that are currently available. Furthermore, the room-temperature sputtering of FTCEs on highly flexible polymer substrates, which are highly compatible with continuous roll-to-roll processing, can facilitate the cost-effective and high-throughput fabrication of large-area electrodes to produce flexible organic solar cells with excellent power-conversion efficiencies.

## Methods

### FTCE fabrication and characterization

ZnO/Cu/ZnO and ZnO/Cu(O)/ZnO, in addition to ZnO/Cu and ZnO/Cu(O), were deposited using multigun magnetron sputtering (A-Tech System Co, Ltd, Flexlab System 100) using 4-inch ZnO and Cu targets (Materion Advanced Materials) at room temperature, without the application of heat during and/or after deposition. The sputtering chamber was initially evacuated to a base pressure of less than 2.0 × 10^−6^ Torr and maintained at 3.0 mTorr during the deposition of ZnO and Cu. The ZnO and Cu films were deposited at a radio frequency power of 200 W (0.53 W cm^−2^) and a direct current power of 50 W, respectively, under a flow of pure Ar (99.9999%), introduced into the chamber at 50 s.c.c.m. The Cu(O) films were deposited by a reactive sputtering process using a gas mixture of Ar and O_2_ (99.999%); a fixed Ar flow rate of 50 s.c.c.m. and a variable O_2_ flow rate were used. The multilayers were subsequently deposited under vacuum conditions. The ITO film was deposited using an ITO target (Advanced Nano Products Co, Ltd) in the sputtering system under the same sputtering conditions used for the ZnO films. The electrodes were deposited on 125-μm-thick PET substrates (Panac Co, Ltd). The oxygen concentrations of the Cu and Cu(O) films were determined using X-ray photoelectron spectroscopy (Escalab 200 R, VG Scientific) measurements with a scan pass energy of 100 eV at the Electronics and Telecommunications Research Institute (Daejeon, Republic of Korea). The highly magnified surface morphologies of the Cu and Cu(O) films were observed using UHR FE-SEM (S-5500, Hitachi Co.) at the Jeonju Center of the Korea Basic Science Institute (Jeonju, Republic of Korea). The surface morphologies were also investigated by tapping mode AFM (NX10, Park System) using a tip with a radius of curvature of less than 10 nm. The surface roughness was averaged from the field of view of at least three different 1 μm × 1 μm surface domains (scan areas) for each sample, using a formula for the root-mean-square height of the surface. The crystallography of the 200-nm-thick Cu and Cu(O) films with different oxygen concentrations was determined in the 2*θ* range of 30–60° using GI-XRD (PANalytical, Empyrean) using Cu K_α_ radiation (1.54 Å) at the Daegu Center of the Korea Basic Science Institute (Daegu, Republic of Korea). The ratio of the Cu_2_O and Cu phases was determined from the Cu_2_O(111) and Cu(111) intensities that were obtained using a single X-ray diffraction measurement for each sample. The thicknesses of the thin films were determined using X-ray reflectivity (Supplementary Fig. 16) at the aforementioned Institute or by surface profiler (P-11, KLA-Tencor) measurements. The total transmittance and reflection spectra were determined in the wavelength range of 300–2,200 nm using ultraviolet-visible-near infrared spectrophotometry (Cary Series, Agilent Technologies). The spectra were measured by excluding the spectra of the PET substrates. The optical absorbance was determined by ascertaining the total transmission and reflection. The sheet resistance was measured using a four-point probe system (MCP-T600, Mitsubishi Chemical Co). The results obtained from three specimens with dimensions of 2.5 cm × 2.5 cm were averaged for each electrode sample. The carrier concentration and mobility were determined through the van der Pauw method using a Hall-effect measurement system (8400 series HMS, Lake Shore Cryotronics Inc.). The ultraviolet radiation test was carried out using a fluorescent lamp (Vilber Lourmat, VL-4.LC) with a power of 4 W at a light wavelength of 365 nm.

### Organic solar cell fabrication and characterization

PTB7-Th (1-material Chemscitech Inc.) and PC_71_BM (Solemme BV) were mixed at a ratio of 8 mg:12 mg in chlorobenzene (1 ml) solution. The mixture was stirred at 50 °C for 12 h, and Diphenyl ether (Sigma-Aldrich) was subsequently added at a volume ratio of 3%. The PTB7-Th:PC71BM solution was filtered using a 0.2-μm poly(tetrafluoroethylene) syringe filter and subsequently spin-coated on the FTCEs at 600–1,200 r.p.m. for 40 s in a glove box. The thickness of the deposited active layer was *ca*. 100 nm with thickness deviations of within 5% (Supplementary Fig. 17). A layer of PEDOT:PSS (Clevios P VP AI 4083) was deposited from a solution mixture of PEDOT:PSS and isopropyl alcohol with a ratio of 1:10. The samples were dried in a glove box at 80 °C for 10 min. Finally, to fabricate the top electrode of the solar cells, using a shadow mask, a Ag metal layer was deposited by thermal evaporation of Ag at 5 × 10^−6^ Torr; the size of the solar cell device was defined using a metal aperture mask with a size of 0.38 cm^2^. The *J−V* characteristics of the solar cells were measured using a Keithley 2400 source measure unit under the illumination of an Air Mass 1.5 Global (AM 1.5G) with an intensity of 100 mW cm^−2^, which was calibrated using a standard PV reference monocrystalline silicon solar cell (2 cm × 2 cm) that was guaranteed by the National Renewable Energy Laboratory (NREL, Colorado, USA). The external quantum efficiency spectra were determined by a solar cell spectral response/QE/IPCE measurement system (PV Measurements Inc.). The series resistance was determined from the slope of the dark current curves, and the IPCE spectra were determined by a quantum efficiency measurement system (Oriel IQE-200) with a monochromator, an optical chopper, a lock-in amplifier, a calibrated silicon photodetector and a 250-W quartz tungsten halogen lamp as the light source. Five samples were characterized and the average results were recorded for each solar cell. The mechanical flexibility of the solar cells fabricated on the PET substrates was determined as a function of the bending radius using an irreversible two-point bending test.

## Additional information

**How to cite this article:** Zhao, G. *et al*. Stable ultrathin partially oxidized copper film electrode for highly efficient flexible solar cells. *Nat. Commun.* 6:8830 doi: 10.1038/ncomms9830 (2015).

## Supplementary Material

Supplementary Information Supplementary Figures 1-17 and Supplementary Tables 1-5.

## Figures and Tables

**Table 1:** Photovoltaic performance of inverted organic solar cells with different FTCEs.

**Electrode type**	***J***_**sc**_ **(mA cm**^−2^)	**Integrated** ***J***_**sc**_ **(mA cm**^−2^)	***V***_**oc**_ **(V)**	**FF (%)**	***R***_**s**_ **(Ω cm**^2^)	**Average PCE (%)**	**Best PCE (%)**
ITO 120 nm/ZnO	17.10±0.34	17.00±0.25	0.78±0.01	51.66±0.59	7.62±0.35	6.91±0.10	7.01
ZnO/Cu 6.5 nm/ZnO	14.65±0.21	14.23±0.34	0.76±0.01	55.59±0.42	6.71±0.12	6.20±0.02	6.22
ZnO/Cu 8.0 nm/ZnO	15.60±0.14	15.50±0.21	0.77±0.01	55.54±0.49	6.21±0.19	6.64±0.10	6.74
ZnO/Cu(O=5.0%) 5.0 nm/ZnO	16.42±0.08	16.33±0.10	0.78±0.01	55.65±0.33	5.11±1.32	7.13±0.05	7.18
ZnO/Cu(O=5.0%) 7.0 nm/ZnO	16.52±0.22	16.47±0.10	0.78±0.01	57.91±0.10	4.82±1.71	7.50±0.09	7.65

FF, fill factor; FTCE, flexible transparent conducting electrode; ITO, indium tin oxide; *J*_sc_, short-circuit current density; PCE, power conversion efficiency; *R*_s_, series resistance; *V*_oc_, open-circuit voltage; ZnO, zinc oxide.

Each parameter was calculated from an average value of five solar cells fabricated on each electrode type. Error bars represent the s.d.
