# Supplementary material for: Stable ultrathin partially oxidized copper film electrode for highly efficient flexible solar cells
Source: Nat Commun. 2015 Nov 5;6:8830. doi: 10.1038/ncomms9830 (PMC4667621; doi:10.1038/ncomms9830)
Supplement: Supplementary Information — Supplementary Figures 1-17 and Supplementary Tables 1-5. [file ncomms9830-s1.pdf]

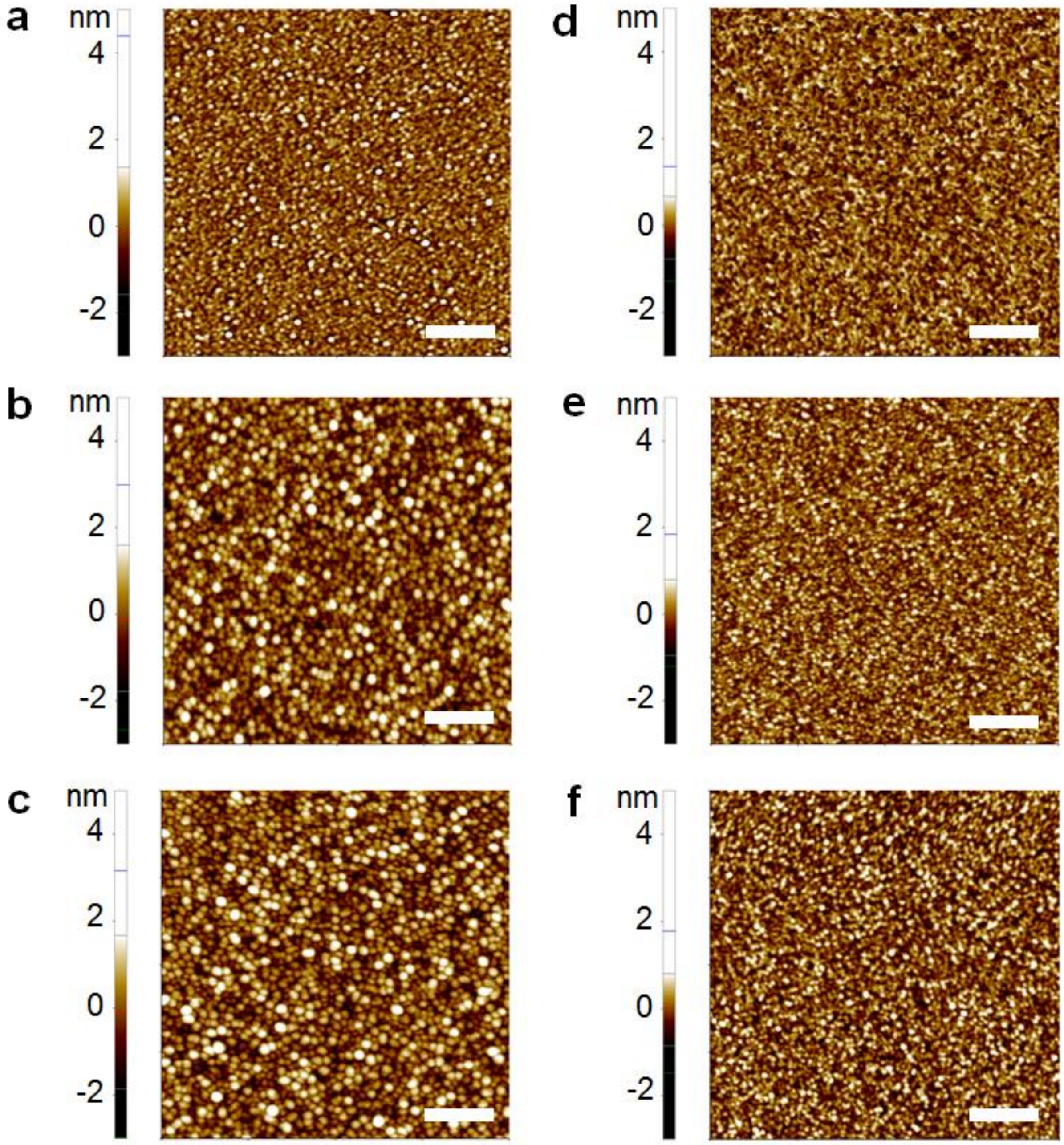

Supplementary Fig. 1 Atomic force microscopy topography images Two-dimensional atomic force microscopy images (with an area of 1 μm × 1 μm) of Cu and Cu(O = 5.0%) films deposited on 20-nm-thick ZnO films during the very early growth stages. Cu films of various thicknesses: (a) 1.5 nm, (b) 2.5 nm, and (f) 5.0 nm; and Cu(O = 5.0%) films of various thicknesses: (d) 1.5 nm, (e) 2.5 nm, and (f) 5.0 nm. The Cu and Cu(O = 5.0%) films were subsequently deposited on the ZnO films by a room-temperature sputtering process, without breaking vacuum. The Cu(O = 5.0%) films were deposited under reactive sputtering conditions using a gas mixture of Ar (50 sccm) and O_2 (0.1 sccm). Scale bar, 200 nm.

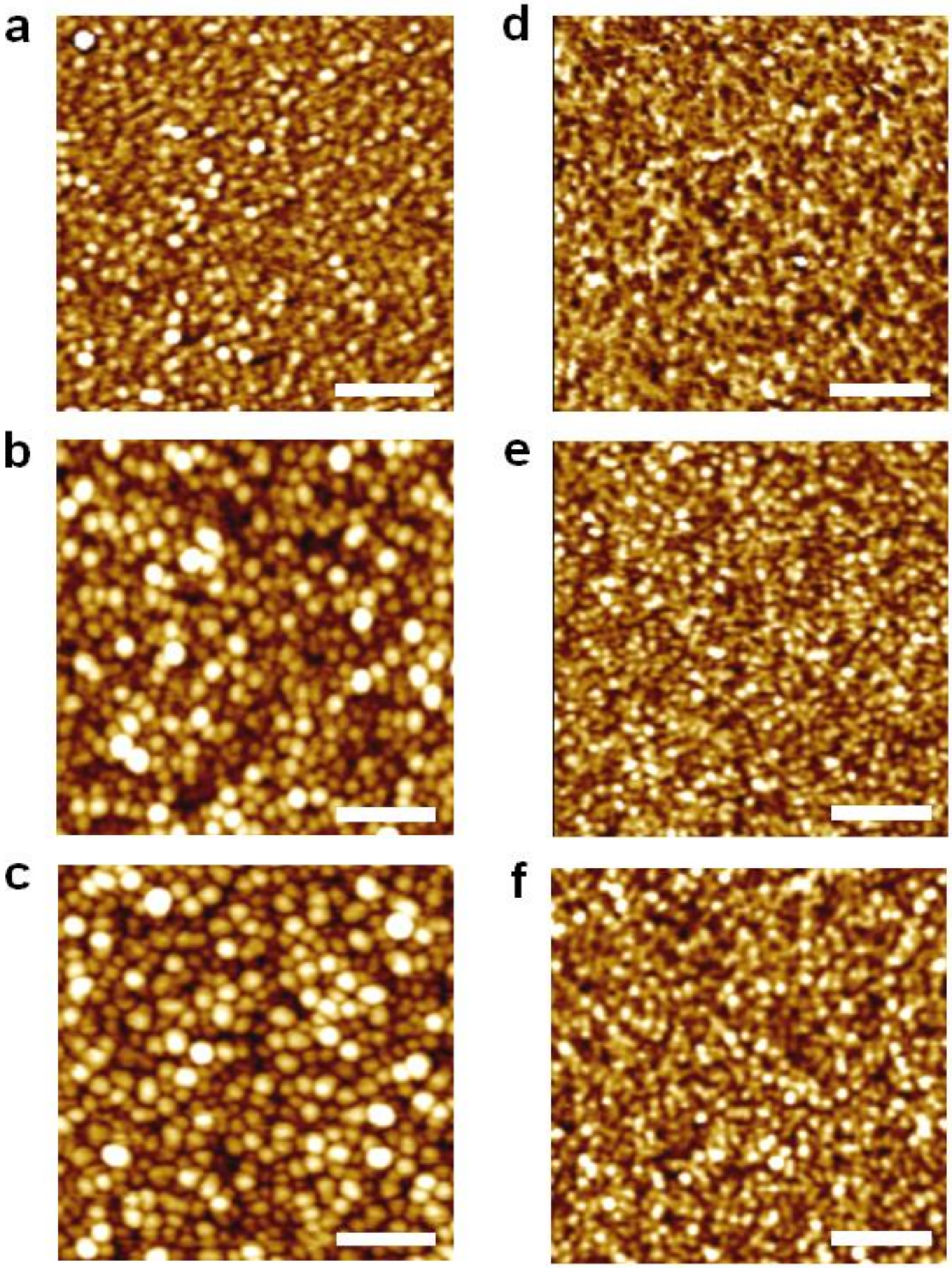

Supplementary Fig. 2 Highly magnified atomic force microscopy topography images Magnified views of atomic force microscopy images corresponding to Supplementary Fig. 1. Cu films of various thicknesses: (a) 1.5 nm, (b) 2.5 nm, and (c) 5.0 nm; and Cu(O = 5.0%) films of various thicknesses: (d) 1. 5nm, (e) 2.5 nm, and (f) 5.0 nm. Scale bar, 100 nm.

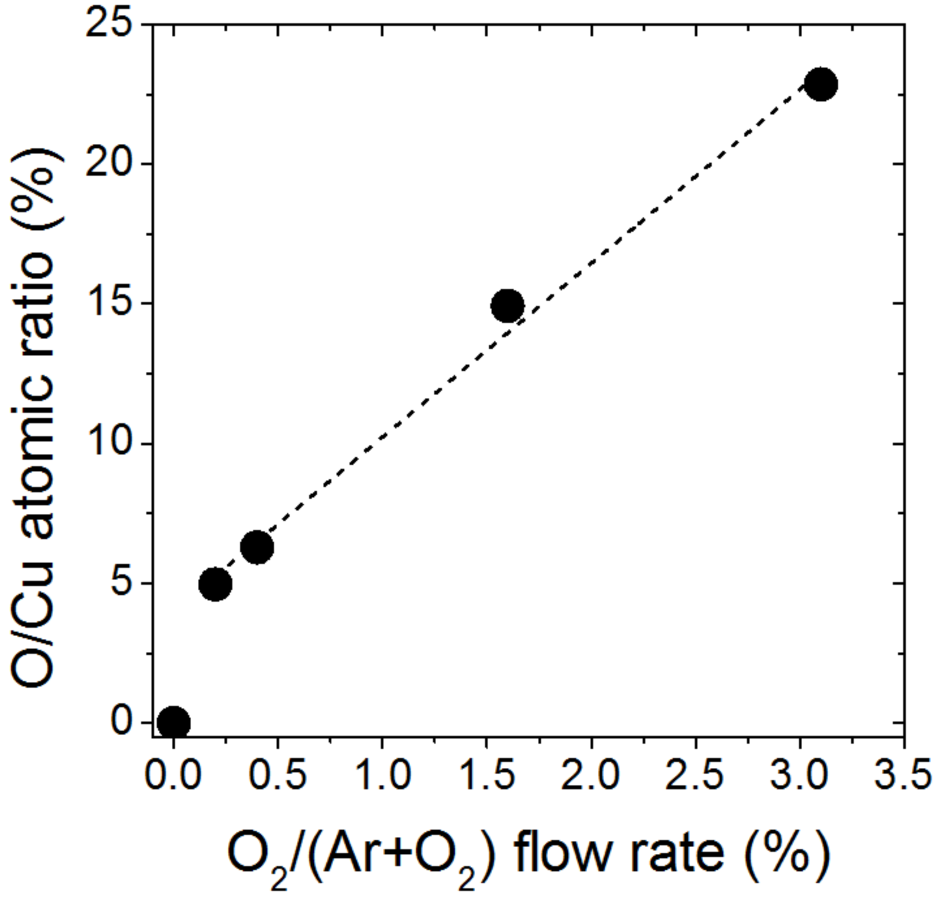

Supplementary Fig. 3 Oxygen control in Cu(O) films Change in the O/Cu atomic ratio of Cu(O) films as a function of the inlet flow rates of O_2 versus the Ar gases used for their reactive sputtering processes, where a fixed Ar flow rate of 50 sccm and a variable O_2 flow rate were used. The O/Cu atomic ratio was evaluated from the Cu(O) films by X-ray photoelectron spectroscopy. The dotted line is added to guide the eyes.

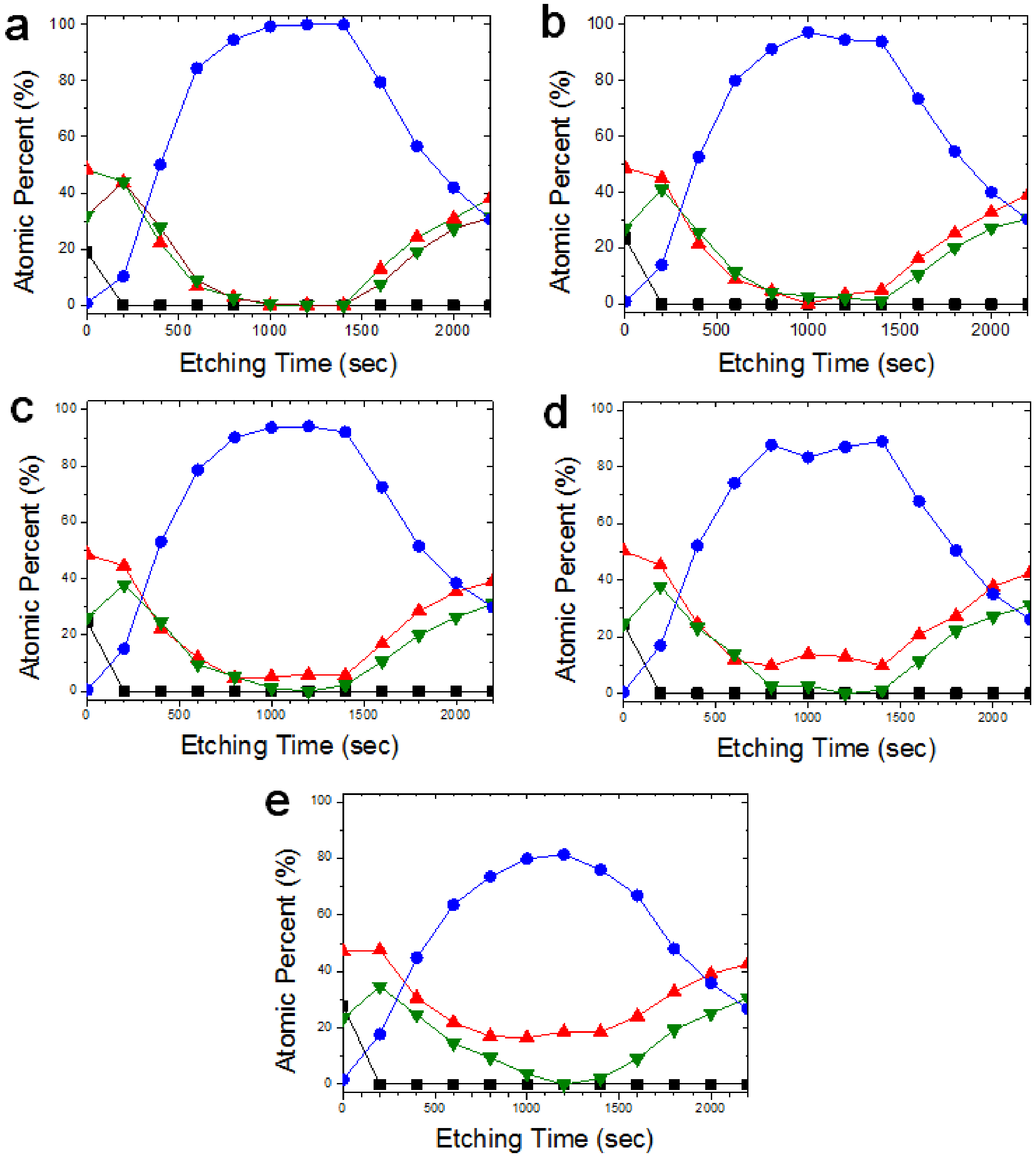

Supplementary Fig. 4 X-ray photoelectron spectroscopy depth profiling Relative atomic concentration of (blue circle) Cu, (red triangle) O, (green inverted triangle) Zn, and (black square) C species in Cu and Cu(O) films, which were deposited under different $O_2/(Ar + O_2)$ flow rates: (a) 0%, (b) 0.2%, (c) 0.4%, (d) 1.6%, and (e) 3.1%. The 20-nm-thick Cu and Cu(O) films were encapsulated between 10-nm-thick ZnO films. The oxidation levels of the films were determined at a constant etching time of 1200 s. The lines are added to guide the eyes.

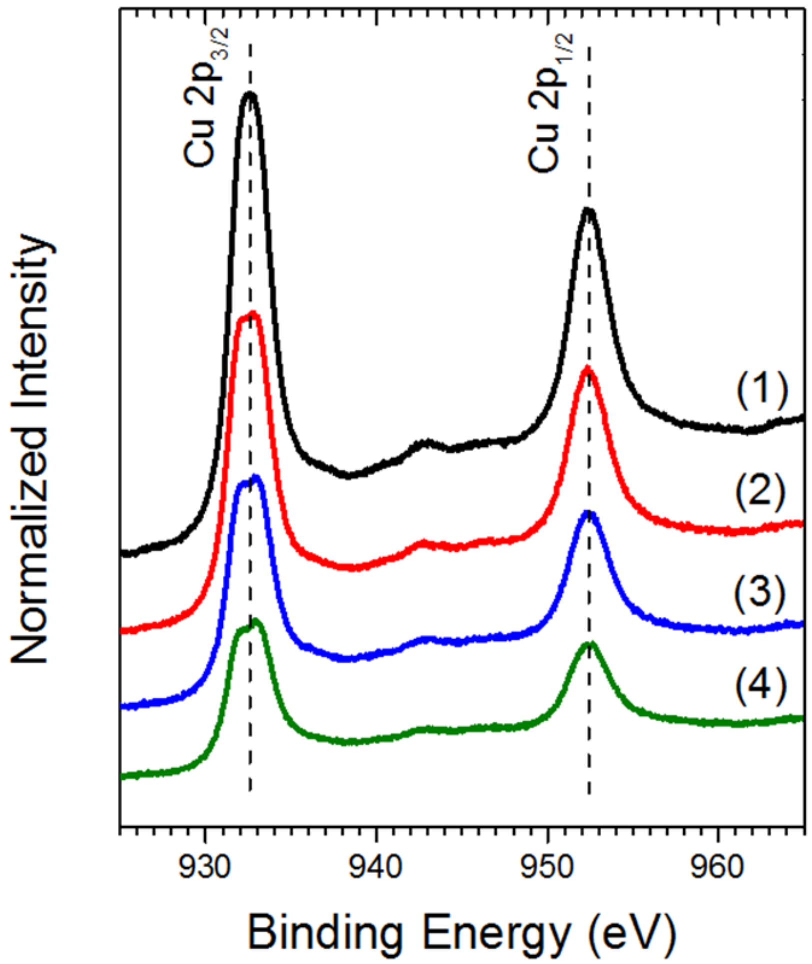

Supplementary Fig. 5 X-ray photoelectron spectra Cu 2p peak spectra of Cu and Cu(O) films with different O /Cu ratios: (1) 0%, (2) 5.0%, (3) 6.3%, and (4) 14.0%, where the spectra were determined at a constant etching time of 1200 s during the depth profiling.

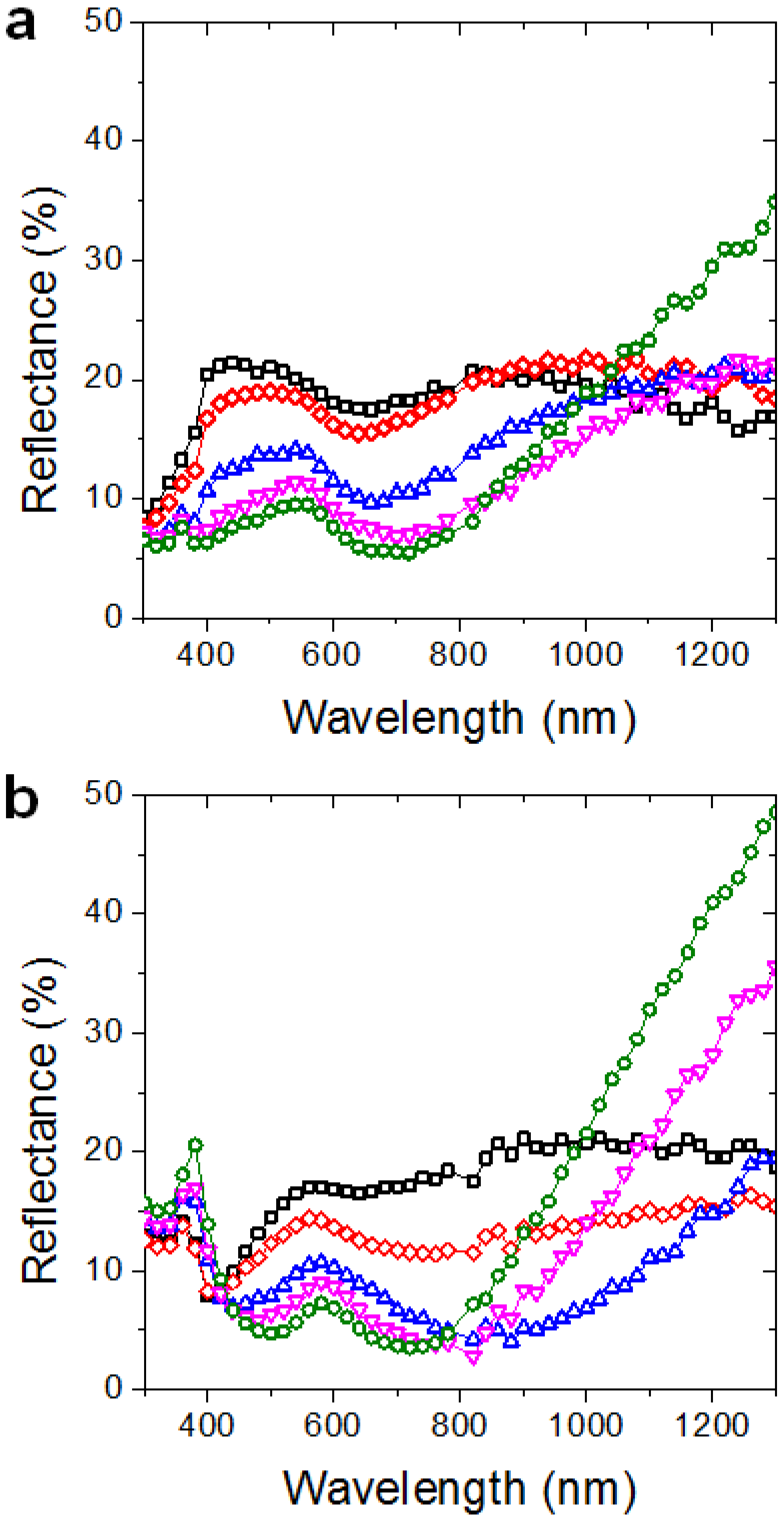

Supplementary Fig. 6 Total reflection spectra Total reflection spectra of (a) ZnO/Cu/ZnO and (b) ZnO/Cu(O = 5.0%)/ZnO FTCEs deposited on polyethylene terephthalate polymer substrates with various Cu and Cu(O) film thicknesses: (black square) 1.5-nm-thick Cu and Cu(O = 5.0%), (red diamond) 2.5-nm-thick Cu and Cu(O), (blue triangle) 5.0-nm-thick Cu and Cu(O), (magenta inverted triangle) 6.5-nm-thick Cu and 7.0-nm-thick Cu(O), (green circle) 8.0-nm-thick Cu and Cu(O) films. The thickness of ZnO films was fixed at 60 nm.

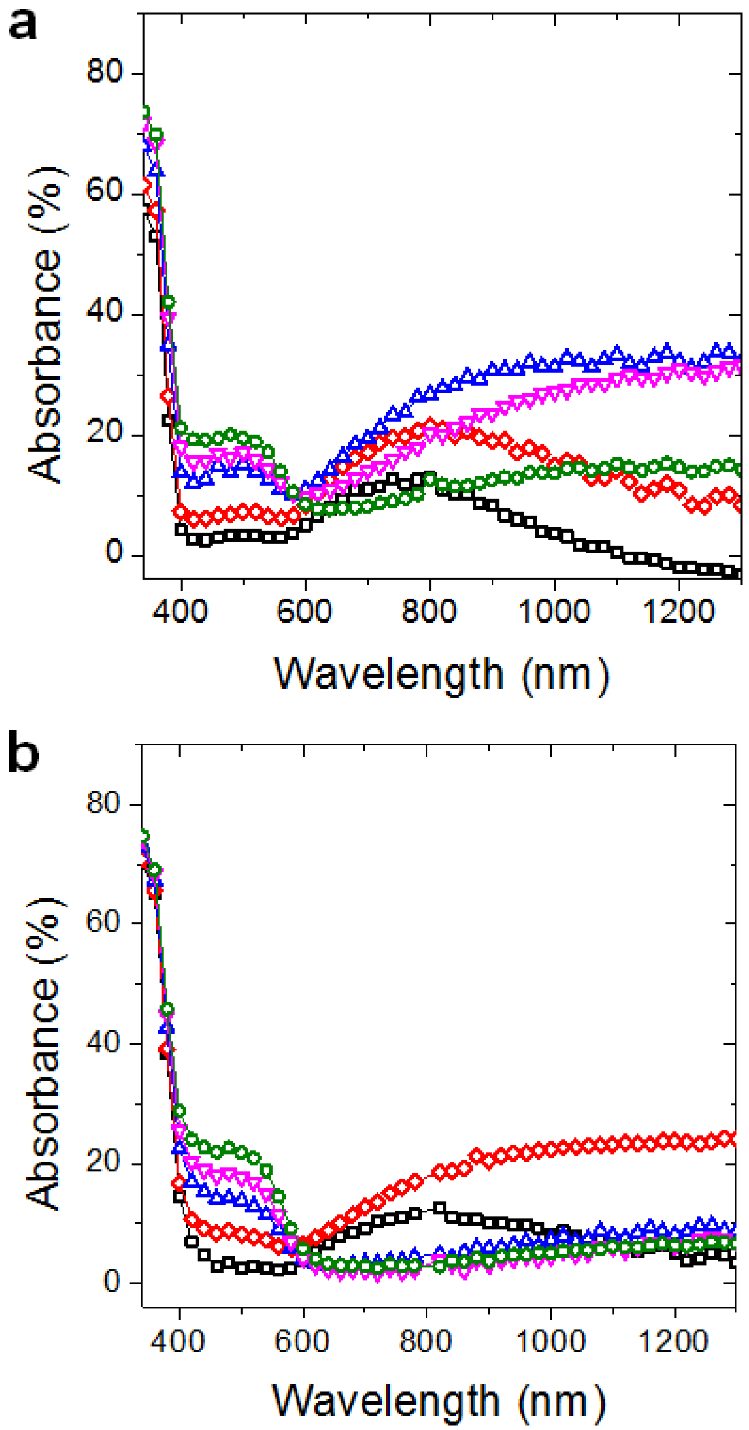

Supplementary Fig. 7 Optical absorbance spectra Absorbance spectra of the (a) ZnO/Cu/ZnO and (b) ZnO/Cu(O = 5.0%)/ZnO FTCEs, corresponding to Supplementary Fig. 6, with various Cu and Cu(O = 5.0%) film thicknesses: (black square) 1.5-nm-thick Cu and Cu(O), (red diamond) 2.5-nm-thick Cu and Cu(O), (blue triangle) 5.0-nm-thick Cu and Cu(O), (magenta inverted triangle) 6.5-nm-thick Cu and 7.0-nm-thick Cu(O), (green circle) 8.0-nm-thick Cu and Cu(O) films. The thickness of ZnO films was fixed at 60 nm. Absorbance was determined by a simple formula, 100–(total transmittance + total reflection).

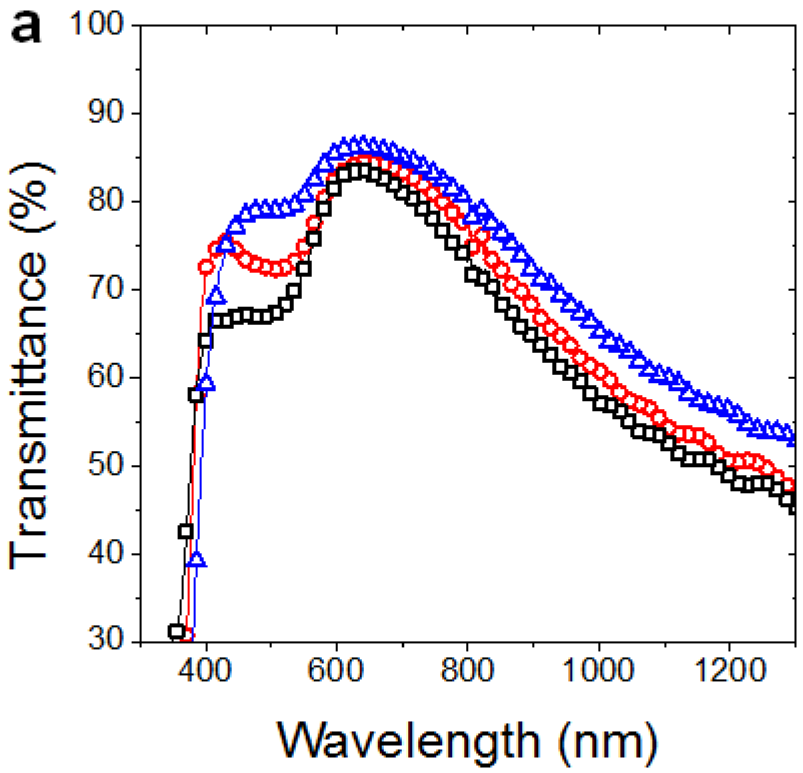

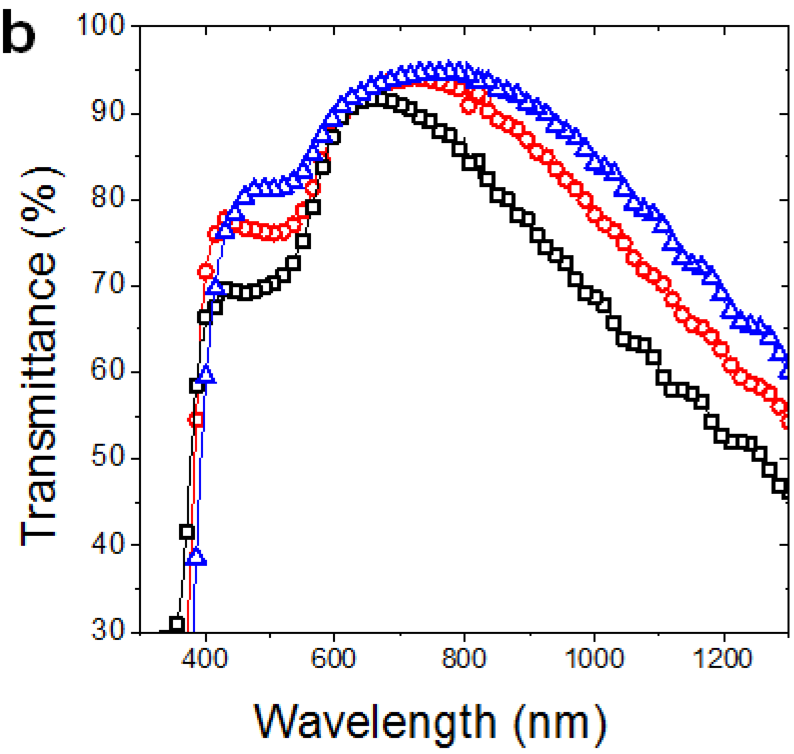

Supplementary Fig. 8 Effects of different ZnO thicknesses on total transmittance. Total transmittances of the (a) ZnO/8-nm-thick Cu/ZnO and (b) ZnO/7.0-nm-thick Cu(O = 5.0%)/ZnO FTCEs with ZnO thicknesses: (black square) 40 nm, (red circle) 60 nm, and (blue triangle) 80 nm.

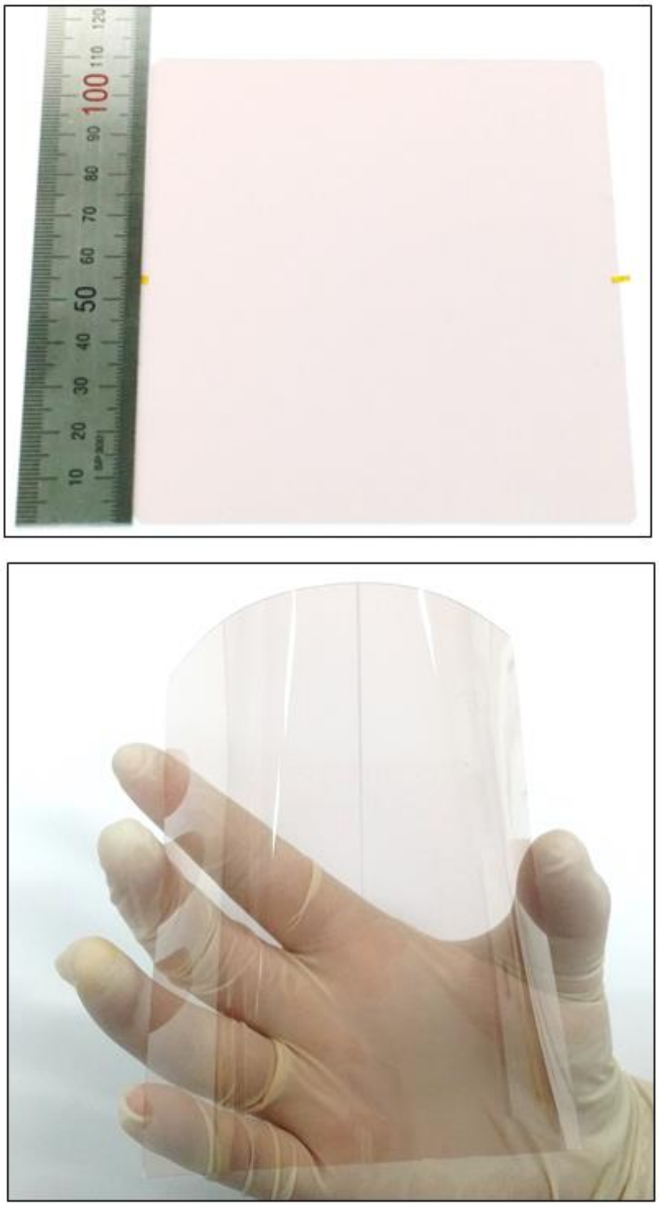

Supplementary Fig. 9 Optical photography of flexible FTCEs Optical images of the 60-nm-thick ZnO/7-nm-thick Cu(O = 5.0%)/60-nm-thick ZnO FTCE deposited on a highly flexible polymer substrate with an enlarged area (11 cm × 11 cm).

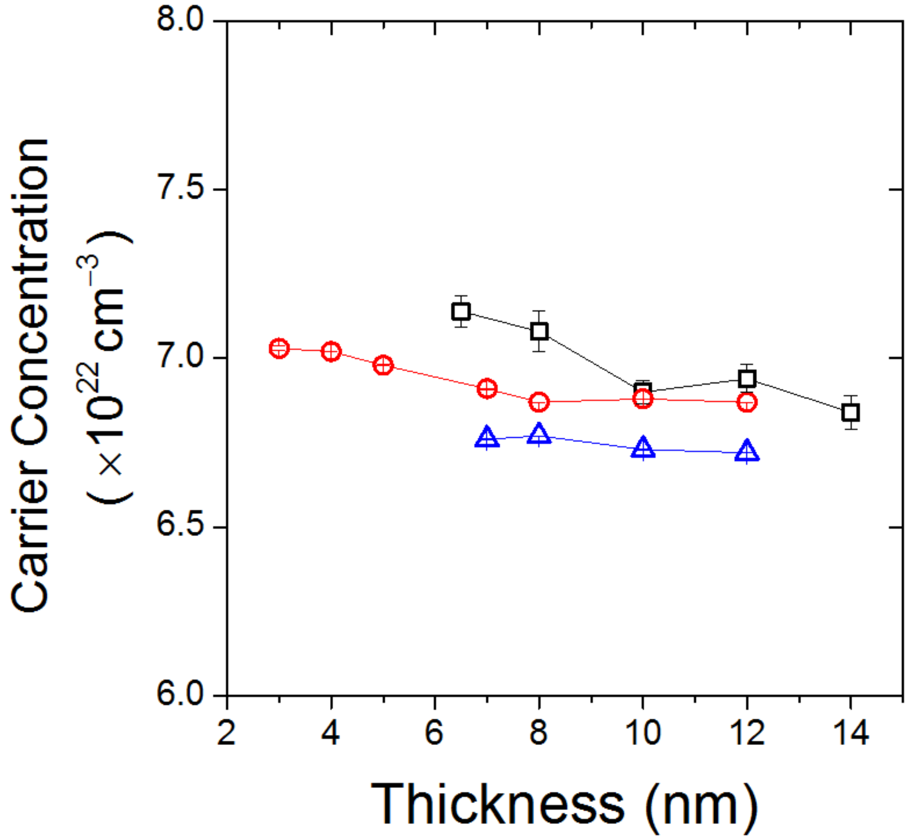

Supplementary Fig. 10 Carrier concentration determination Change in carrier concentrations of (black square) ZnO/Cu/ZnO, (red circle) ZnO/Cu(O = 5.0%)/ZnO, and (blue triangle) ZnO/Cu(O = 6.3%)/ZnO FTCEs using constant 60-nm-thick ZnO films with various Cu and Cu(O) film thicknesses corresponding to Fig. 3f. Average carrier concentration values were determined from three different measurements for each sample. Error bars indicate the standard deviation. The lines are added to guide the eyes.

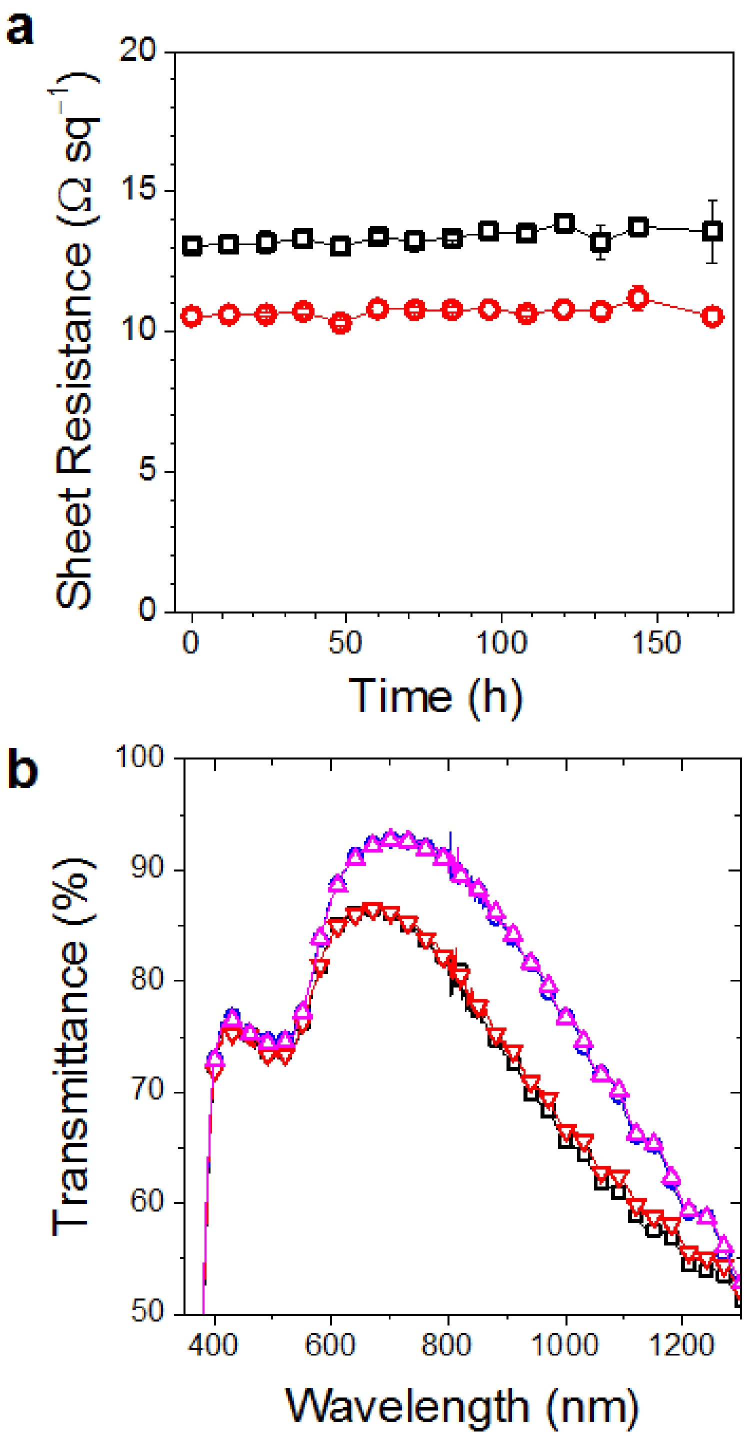

Supplementary Fig. 11 Resistance against UV exposure (a) Change in the sheet resistance of (black square) 60-nm-thick ZnO/8-nm-thick Cu/60-nm-thick ZnO and (red circle) 60-nm-thick ZnO/7-nm-thick Cu(O = 5.0%)/60-nm-thick ZnO with long-term exposure to UV radiation for 170 h. Average sheet resistance values were determined from three different measurements for each sample. Error bars indicate standard deviation. The lines are added to guide the eyes. (b) Changes in the total transmittance of the ZnO/8-nm-thick Cu/ZnO FTCE, (black square) before and (red inverted triangle) after UV exposure, and the ZnO/7-nm-thick Cu(O =5.0%)/ZnO FTCE, (blue circle) before and (magenta triangle) after UV exposure.

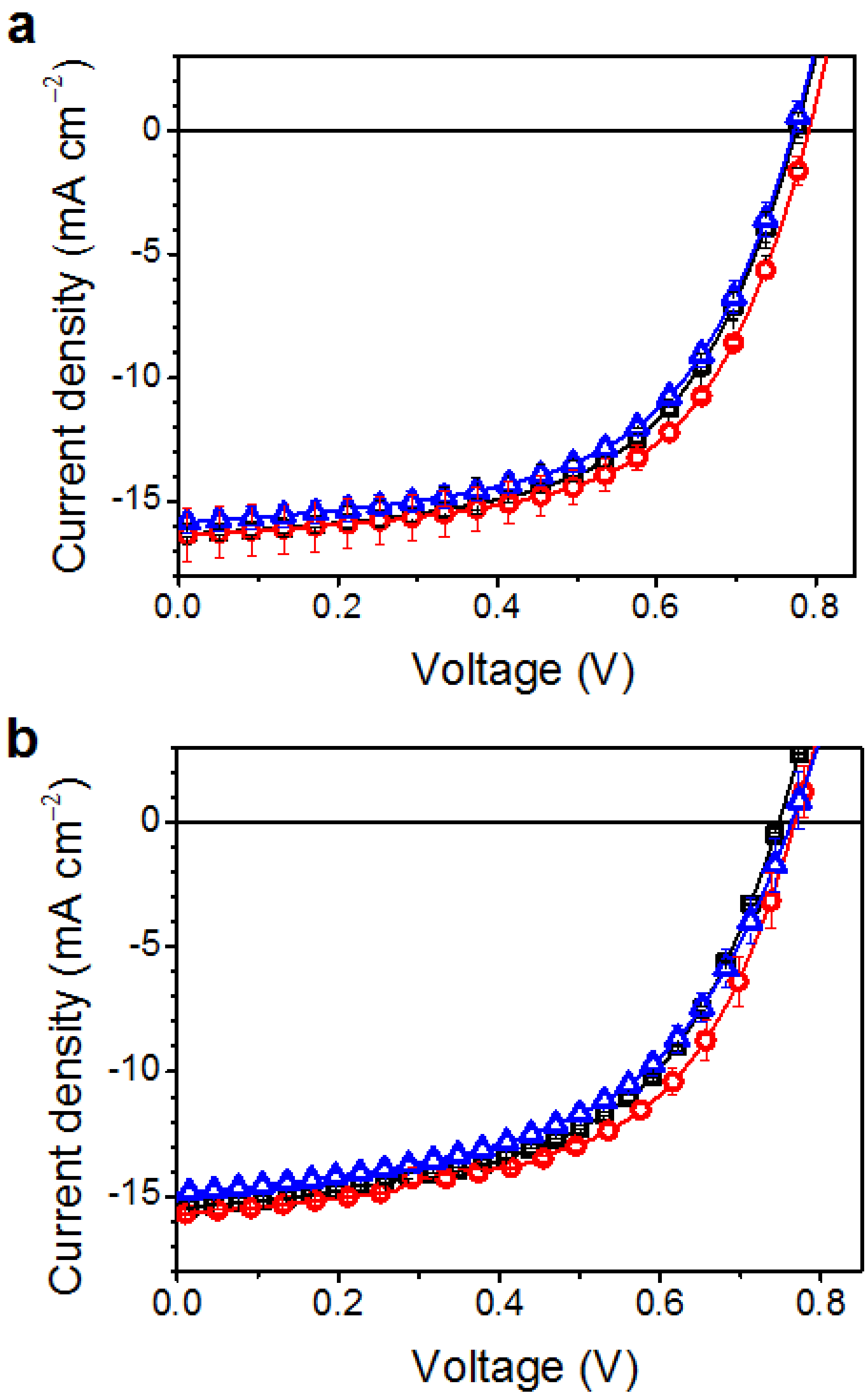

Supplementary Fig. 12 Effect of different ZnO thicknesses on *J*–*V* characteristics Comparison of the *J*–*V* characteristics of inverted organic solar cells for different ZnO thicknesses: (black square) 40 nm, (red circle) 60 nm, and (blue triangle) 80 nm, used in the (a) ZnO/8-nm-thick Cu/ZnO and (b) ZnO/7-nm-thick Cu(O = 5.0%)/ZnO FTCEs. Error bars represent the standard deviation (n =5).

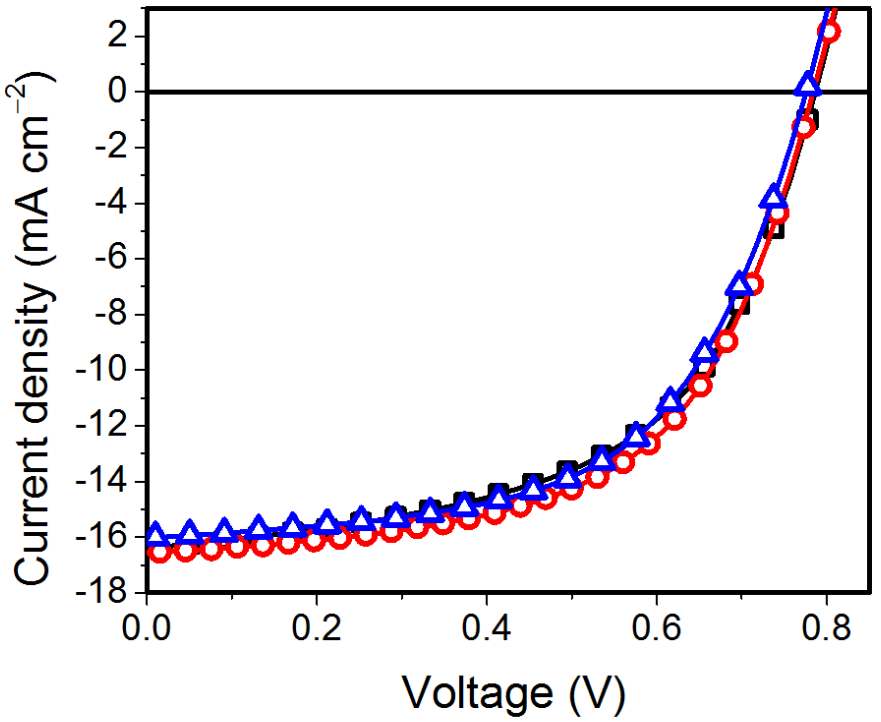

Supplementary Fig. 13 Effect of different photoactive layer thicknesses on *J*–*V* characteristics Comparison of the *J*–*V* characteristics of inverted organic solar cells comprising the 60-nm-thick ZnO/7-nm-thick Cu(O = 5.0%)/60-nm-thick ZnO FTCE, with different photoactive layer thicknesses: (black square) 70 nm, (red circle) 100 nm, and (blue triangle) 130 nm.

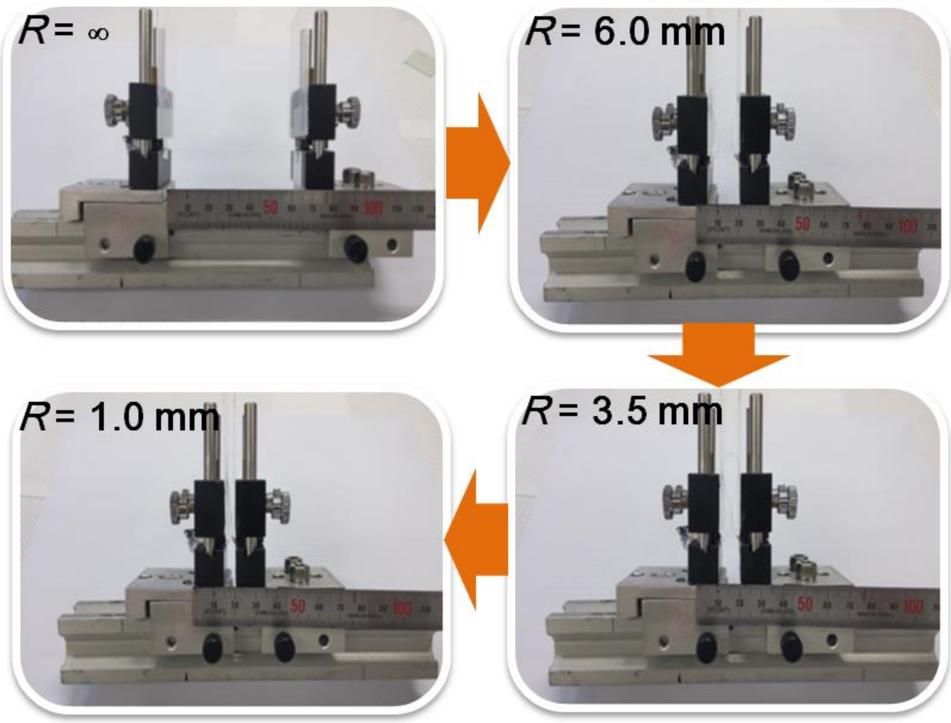

Supplementary Fig. 14 Mechanical flexibility test of FTCEs Photographs of an irreversible two-point bending test as a function of bending radius. The FTCEs deposited on 125-μm-thick polyethylene terephthalate substrates were exposed to compressive stress induced by the bending.

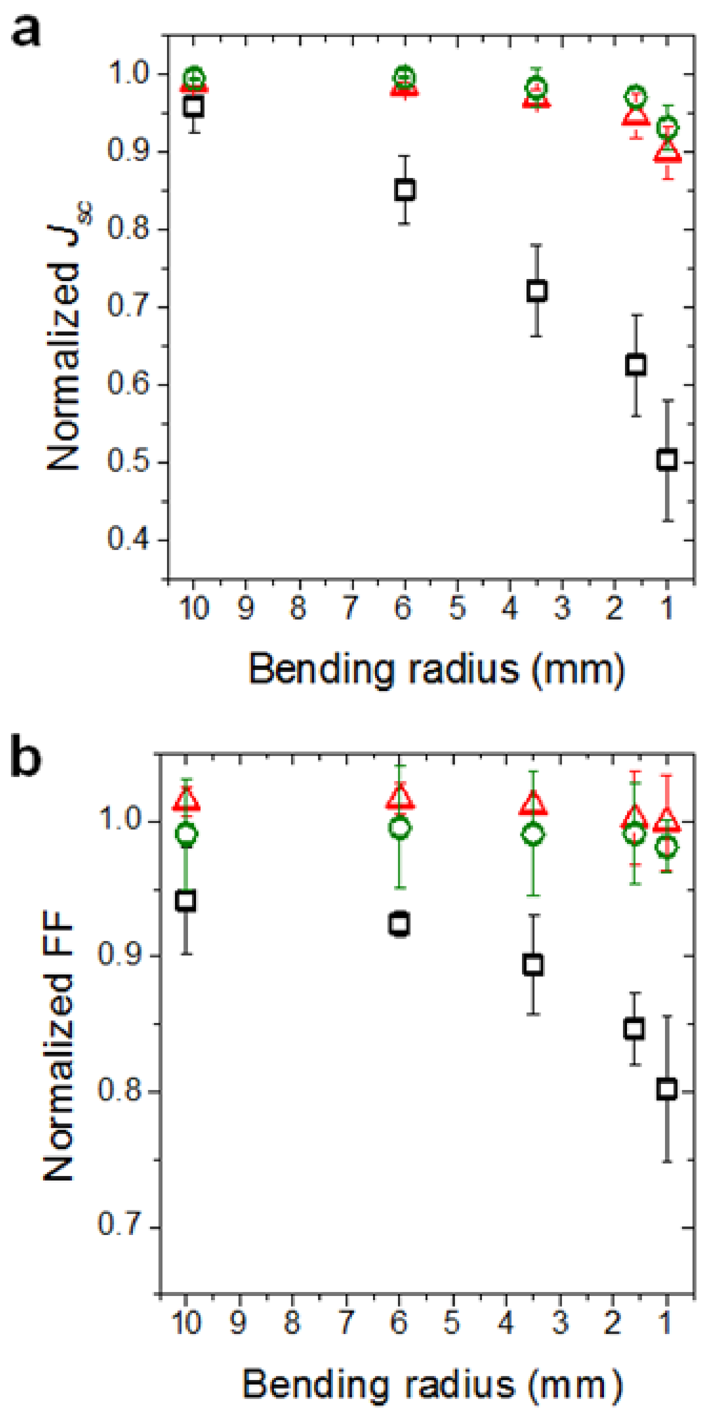

Supplementary Fig. 15 Change in photovoltaic performance with mechanical bending Change in the (a) short-circuit current density (J_{sc}) and (b) fill factor (FF) values measured for flexible solar cells as a function of bending radius during compressive bending, normalized to their initial values. Three different FTCEs were examined in the solar cells and they were (black square) 120-nm-thick ITO/50-nm-thick ZnO, (green circle) 60-nm-thick ZnO/7-nm-thick Cu(O = 5.0%)/60-nm-thick ZnO, and (red triangle) 60-nm-thick ZnO/8-nm-thick Cu/60-nm-thick ZnO. Each parameter was calculated from an average value of 5 solar cell samples fabricated on each FTCE type. Error bars represent the standard deviation.

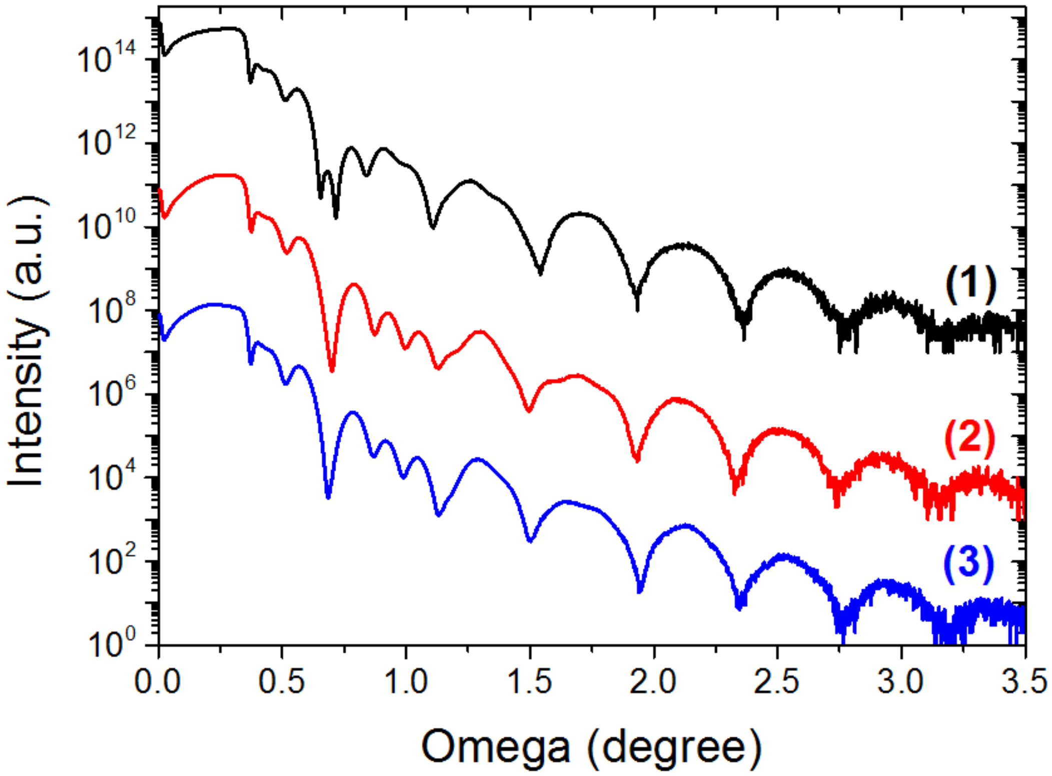

Supplementary Fig. 16 X-ray reflectivity characterization. X-ray reflectivity spectra measured for (1) ZnO/8-nm-thick Cu/ZnO, (2) ZnO/7-nm-thick Cu(O = 5.0%)/ZnO, and (3) ZnO/Cu(O = 6.3%)/ZnO FTCEs.

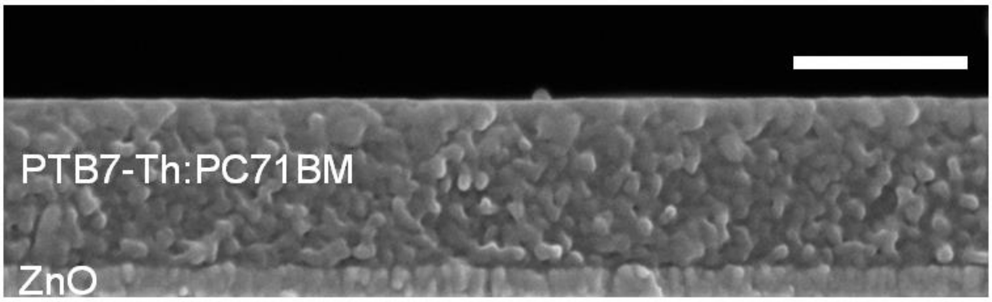

Supplementary Fig. 17 Thickness uniformity of photoactive layer. Cross-sectional FE-SEM image showing the thickness uniformity of a 100-nm-thick photoactive layer coated on a ZnO layer. Scale bar, 100 nm.

Supplementary Table 1 Crystallographic properties of Cu and Cu(O) films

O/Cu (at%)	Cu(111)			Cu_2O(111)		
	2θ (°)	FWHM (°)	Intensity (a.u.)	2θ (°)	FWHM (°)	Intensity (a.u.)
0	43.336	0.423	290476			
5.0	43.308	0.469	108366	36.550	1.390	877
6.3	43.322	0.517	50586	36.540	1.470	1151
14.9	43.293	0.957	12335	36.571	1.530	2508

Change in the crystallographic properties of Cu(111) and Cu_2O(111) due to oxygen inclusion in Cu films corresponding to those shown in Figs. 2k and 2l.

Supplementary Table 2 Sheet resistances of the FTCEs

Electrode type	Thickness (nm)	R_{sheet} (Ω sq^{-1})
ZnO/Cu/ZnO	60/2.5/60	2.9×10^7
	60/5.0/60	1.7×10^3
	60/8.0/60	15.7
ZnO/Cu(O = 5.0%)/ZnO	60/2.5/60	2.1×10^3
	60/5.0/60	21.5
	60/7.0/60	13.1
ZnO/Cu(O = 6.3%)/ZnO	60/2.5/60	1.5×10^2
	60/5.0/60	20.5
	60/7.0/60	14.5

Changes in the sheet resistance (R_{sheet}) of ZnO/Cu/ZnO, ZnO/Cu(O = 5.0%)/ZnO, and ZnO/Cu(O = 6.3%)/ZnO FTCEs with thicknesses of Cu and Cu(O) films, corresponding to Fig. 3b.

Supplementary Table 3 Figure of merits of the FTCEs

Electrode type	Thickness (nm)	$T_{avg,\ \lambda = 400–800\ nm}$ (%)	R_{sheet} ($\Omega\ sq^{-1}$)	FoM ($10^{-3}\ \Omega^{-1}$)
ZnO/Cu/ZnO	60/5.0/60	71.8	1723.3	0.021
	60/6.5/60	76.8	42.1	1.70
	60/8.0/60	79.8	15.7	6.65
ZnO/Cu(O = 5.0%)/ZnO	60/5.0/60	84.1	21.5	8.21
	60/7.0/60	84.7	13.1	14.47
	60/8.0/60	83.2	9.0	17.66
ITO	120	85.8	66.1	3.27

Comparison of the average transmittance in the visible spectral range (T_{avg}), sheet resistance (R_{sheet}), and figure of merit (FoM) of ITO, ZnO/Cu/ZnO, and ZnO/Cu(O = 5.0%)/ZnO FTCEs.

Supplementary Table 4 Change in photovoltaic performance parameters with ZnO thickness

Electrode type	J_{sc} (mA cm^{-2})	V_{oc} (V)	FF (%)	R_s (Ω cm^2)	Avg. PCE (%)	Best PCE (%)
ZnO 40 nm/Cu 8.0 nm/ZnO 40 nm	15.40±0.05	0.75±0.01	54.06±0.26	6.95±0.17	6.21±0.05	6.26
ZnO 60 nm/Cu 8.0 nm/ZnO 60 nm	15.60±0.14	0.77±0.01	55.54±0.49	6.21±0.19	6.64±0.10	6.74
ZnO 80 nm/Cu 8.0 nm/ZnO 80 nm	14.64±0.20	0.77±0.01	52.97±1.07	8.02±0.11	5.94±0.01	5.95
ZnO 40 nm/Cu(O=5.0%) 7.0 nm/ZnO 40 nm	16.31±0.39	0.77±0.01	57.10±0.82	4.92±0.16	7.20±0.13	7.33
ZnO 60 nm/Cu(O=5.0%) 7.0 nm/ZnO 60 nm	16.52±0.22	0.78±0.01	57.91±0.10	4.82±1.71	7.50±0.09	7.65
ZnO 80 nm/Cu(O=5.0%) 7.0 nm/ZnO 80 nm	15.83±0.42	0.77±0.01	56.86±0.80	6.06±0.28	6.93±0.13	7.06

Photovoltaic performance of organic solar cells fabricated using ZnO/Cu/ZnO and ZnO/Cu(O = 5.0%)/ZnO FTCEs with different ZnO thicknesses, averaged over 5 specimens. Errors represent the standard deviation.

J_{sc}, short-circuit current density; V_{oc}, open-circuit voltage; FF, fill factor; R_s, series resistance; PCE, power conversion efficiency.

Supplementary Table 5 Changes in solar cell performance with photoactive layer thickness

Electrode type	Active layer thickness (nm)	J_{sc} (mA cm^{-2})	V_{oc} (V)	FF (%)	R_s (Ω cm^2)	Avg. PCE (%)	Best PCE (%)
ZnO/Cu(O=5.0%)/ZnO	70	16.27±0.21	0.77±0.01	55.86±0.87	5.55±0.17	7.01±0.08	7.09
ZnO/Cu(O=5.0%)/ZnO	100	16.52±0.22	0.78±0.01	57.91±0.10	4.82±1.71	7.50±0.09	7.65
ZnO/Cu(O=5.0%)/ZnO	130	16.05±0.39	0.78±0.01	57.47±0.56	5.02±0.10	7.21±0.08	7.29

Changes in the photovoltaic performance parameters of organic solar cells using the 60-nm-thick ZnO/7.0-nm-thick Cu(O = 5.0%)/60-nm-thick ZnO FTCE with photoactive layer thicknesses, averaged over 5 specimens. Errors represent the standard deviation.
J_{sc}, short-circuit current density; V_{oc}, open-circuit voltage; FF, fill factor; R_s, series resistance; PCE, power conversion efficiency.
